# Predicting Catalyst Performance From Pt/CeO_2_ Redispersion Kinetics

**DOI:** 10.1002/anie.4743891

**Published:** 2026-07-26

**Authors:** Florian Maurer, Agustin Salcedo, Maria Casapu, David Loffreda, Arik Beck, Paolo Dolcet, Mimoun Aouine, Thierry Epicier, Stephane Loridant, Philippe Vernoux, Carine Michel, Jan‐Dierk Grunwaldt

**Affiliations:** ^1^ Institute for Chemical Technology and Polymer Chemistry (ITCP) Karlsruhe Institute of Technology (KIT) Karlsruhe Germany; ^2^ LCH, UMR 5182 CNRS, ENS De Lyon, Université Claude Bernard Lyon 1 Lyon France; ^3^ Institute of Catalysis Research and Technology (IKFT) Karlsruhe Institute of Technology (KIT) Eggenstein‐Leopoldshafen Germany; ^4^ Department of Chemical Sciences University of Padova Padova Italy; ^5^ Université Lyon 1, CNRS Ircelyon, UMR 5256 Villeurbanne France; ^6^ Matériaux, Ingénierie Et Science, UMR 5510 CNRS, INSA De Lyon, Université De Lyon Villeurbanne France

**Keywords:** catalyst deactivation, ceria cubes, DFT, ETEM, noble metal redispersion, platinum, structural kinetics

## Abstract

Quantitative description of the structural dynamics of nanoparticles by kinetic data is challenging but would represent a significant knowledge leap, as this would enable the forecast of their properties, e.g., in catalysis. A striking example in catalysis is the redispersion of Pt nanoparticles into single atoms in Pt/CeO_2_‐based catalysts. In this work, we combined environmental transmission electron microscopy (ETEM) measurements with catalytic data to monitor the individual decay of Pt nanoparticles on defined CeO_2_ nanocubes. Supported by density functional theory modeling, this provides unprecedented insight into their dynamic behavior, including kinetics. We observed that the rate of noble metal redispersion is strongly dependent on the local structural environment: the presence of other nearby nanoparticles and heterogeneities on the CeO_2_ surface reduced the redispersion rate. Independent of the initial particle size and local environment, the particle volume decreases linearly in time, indicating a constant flux of Pt atoms from the nanoparticles. These findings at the atomic scale were correlated to the observed changes in the integral catalytic performance, allowing a first prediction of the catalyst activity based on the redispersion process and demonstrating how atomic‐scale kinetic insights can be correlated to macroscopic effects.

## Graphical abstract

Unravelling kinetics of dynamic structural changes in catalysts is the key to predicting their catalytic properties. ETEM experiments reveal a continuous decrease in Pt particle size on cube‐shaped CeO_2_ under oxidizing conditions, changing from large nanoparticles to highly dispersed species. Correlating these time‐dependent structural changes with catalytic activity enables predicting the performance of Pt‐based catalysts over their lifetime.

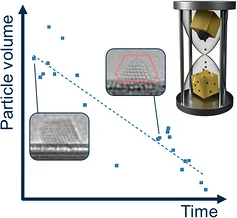

## 1 Introduction

Predicting the evolution of the properties for nano‐sized functional materials under reaction conditions is fundamental for understanding their fate during processes like catalysis. In addition, this opens up the knowledge‐based operation, including re‐establishment of the most active structures, e.g., specific (re)activation strategies. For this purpose, qualitative observations of structural phenomena are not sufficient. Quantitative kinetic data describing the dynamics of nanoparticles [1] (NPs) are essential [2, 3, 4, 5] and would represent a significant knowledge leap for predicting their functionality. For instance, the dynamics of platinum group metal (PGM) NPs supported on reducible oxides are known to strongly affect the catalytic activity depending on their formation or redispersion under reaction conditions [6, 7, 8]. As supported Pt NPs are irreplaceable for many applications, including fuel cells [9, 10], catalytic synthesis of fine chemicals [11, 12, 13], catalytic sensors, or for clean air purposes [14, 15, 16, 17], an accurate quantification of their structural dynamics is key.

During recent years, the strong interaction of PGMs with f‐group metal oxides such as CeO_2_ was in the focus of intense research [18, 19, 20]. In the case of Pt, such a property is beneficial for the catalytic activity [7, 21, 22, 23, 24], and also prevents the sintering of the noble metal under oxygen‐rich reaction conditions [6, 25, 26]. However, this noble metal‐support affinity can be detrimental to the stability of Pt NPs, as their redispersion into cationic single atoms may result in a pronounced catalyst deactivation [6, 27, 28]. Hence, forecasting and controlling the noble metal (NM) nanoparticle redispersion is a highly promising strategy to maintain a high catalytic activity on all scales [29].

Recently, we reported possible approaches for promoting the noble metal agglomeration to sub‐nanometer Pt and Pd clusters under CO and CH_4_ oxidation conditions [27, 30, 31]. In this regard, increasing the surface noble metal concentration prevents the complete redispersion to NM single sites. This can be achieved by tuning the ratio of NM loading to the CeO_2_ surface area or by using mixtures of CeO_2_ and other metal oxide supports, which foster only a local increase of the NM concentration [31, 32]. Alternatively, different catalyst activation/reactivation strategies are considered to generate reduced NPs or clusters from a highly dispersed NM state. Commonly, reductive treatments are used, which are energy‐intense and should be applied as infrequently as possible over the lifetime of a catalyst or in general functional materials [33, 34, 35].

Despite the discussion of noble metal redispersion in recent studies [6, 27, 28, 36, 37, 38, 39, 40], there is still a lack of knowledge about the atom detachment mechanism and underlying kinetics. Obtaining this information would enable the derived experimental data to be correlated with first‐principle calculations [41, 42], and consequently, the time and duration of the catalyst regeneration processes to be estimated more precisely. However, conducting such kinetic studies is extremely challenging and is often not easily accessible through conventional characterization methods [43]. For nearly all previous studies, the noble metal redispersion was evaluated only indirectly, and often by post‐mortem analysis of differently treated samples. Commonly used in situ/operando bulk techniques, such as Raman spectroscopy, X‐ray photon spectroscopy (XPS), X‐ray absorption spectroscopy (XAS), or X‐ray diffraction (XRD), are generally not suitable to estimate these changes at the atomic scale in heterogeneous nanomaterials and applied catalysts.

To overcome these limitations, in situ and environmental electron transmission microscopy (ETEM) [44] have demonstrated great potential [4, 45, 46, 47]. Applied [30, 32, 33, 48] but also well‐defined [44, 49] NM‐CeO_2_‐based catalysts have been investigated under reductive or oxidative gas atmospheres in a broad temperature range. While certain processes like surface reconstructions [5] were already quantitatively investigated, the nanoparticle redispersion was not adequately captured, and no kinetic data have been derived so far. In this study, we systematically estimate the redispersion mechanism and determine the kinetic parameters for selected Pt NPs supported on well‐defined cube‐shaped CeO_2_ under oxidizing conditions. By utilizing CeO_2_ cubes, surfaces supporting Pt nanoparticles were aligned parallel to the electron beam, which allowed monitoring their cross sections over time. Density functional theory (DFT) calculations were used to support the interpretation of the experimental data by analyzing structural changes upon oxidation of the NPs, and verifying the thermodynamic feasibility of the mechanism proposed. Finally, the trends in structural dynamics at the atomic level were correlated to the integral catalytic activity, thus bridging the pressure and scale gaps.

## 2 Results and Discussion

### 2.1 Material Synthesis

**FIGURE 1:**
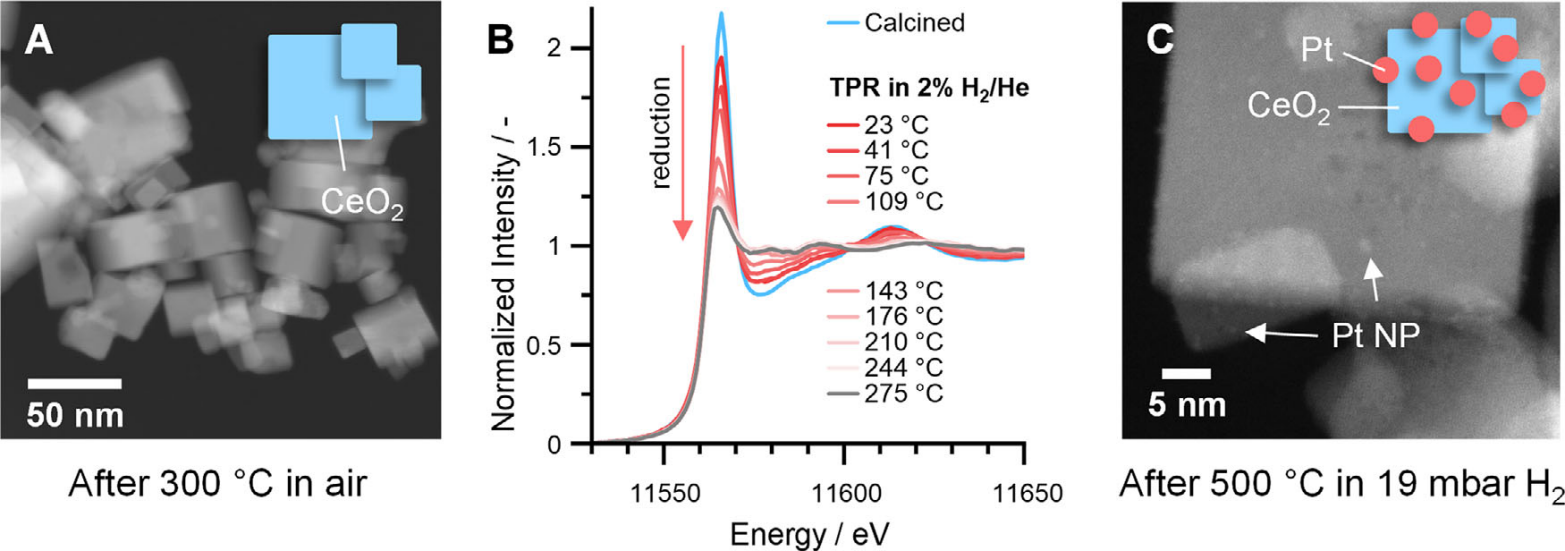
The HAADF‐STEM image (A) shows the as‐prepared CeO_2_ cubes. After Pt impregnation, the sample was reduced in 2% H_2_/He to form nanoparticles, as followed by XANES at Pt L_3_ edge (B); the formation of NPs is further confirmed by the bright shadows in the HAADF‐STEM image (C).

Cube‐shaped CeO_2_ with a surface area of 23 m^2^/g was hydrothermally synthesized according to the procedure developed by Mai et al., [50]. The ∼10–50 nm CeO_2_ cubes displayed in Figure 1A (further TEM images in Figure S1) were then impregnated with a Pt(NH_3_)_4_(NO_3_)_2_ solution until the desired loading of 1.0 wt.% Pt was achieved. After drying in static air, a calcination step at 300°C was applied. As no Pt particles were visible in the as‐prepared Pt/CeO_2_ catalyst, it is assumed that the noble metal is present as highly dispersed species (cf. Figure S2). Starting from this state, homogenously distributed Pt NPs can be generated by reductive treatments [16, 33]. For the present sample, this structural change was validated by in situ X‐ray absorption near‐edge structure (XANES, Figure 1B) spectroscopy measurements during catalyst reduction in 2% H_2_/He. The results of the corresponding linear combination fitting (LCF, Figure S3) indicate that Pt^4+^ is the major species (80%) at the start of the experiment. Up to 150°C, a fast reduction process was observed, and at 250°C, only reduced Pt was present. This reductive treatment of the as‐prepared sample was repeated in the environmental electron microscope (500°C, 17 mbar H_2_, 60 min) to in situ produce Pt NPs. Although approx. the same H_2_ partial pressure was maintained, a higher reduction temperature was applied to compensate for a potentially lower local H_2_ pressure resulting from its increased diffusivity. High‐angle angular darkfield transmission electron microscopy (HAADF–STEM) images (Figure 1C) collected afterwards at 200°C display the formation of reduced Pt NPs of 1.3 nm ± 0.3 nm (particle radius). In the following sections, the morphology and particle size of Pt NPs are further investigated by ETEM measurements under oxidizing conditions.

### 2.2 Direct Observation of the Redispersion of Pt NPs on CeO_2_

**FIGURE 2:**
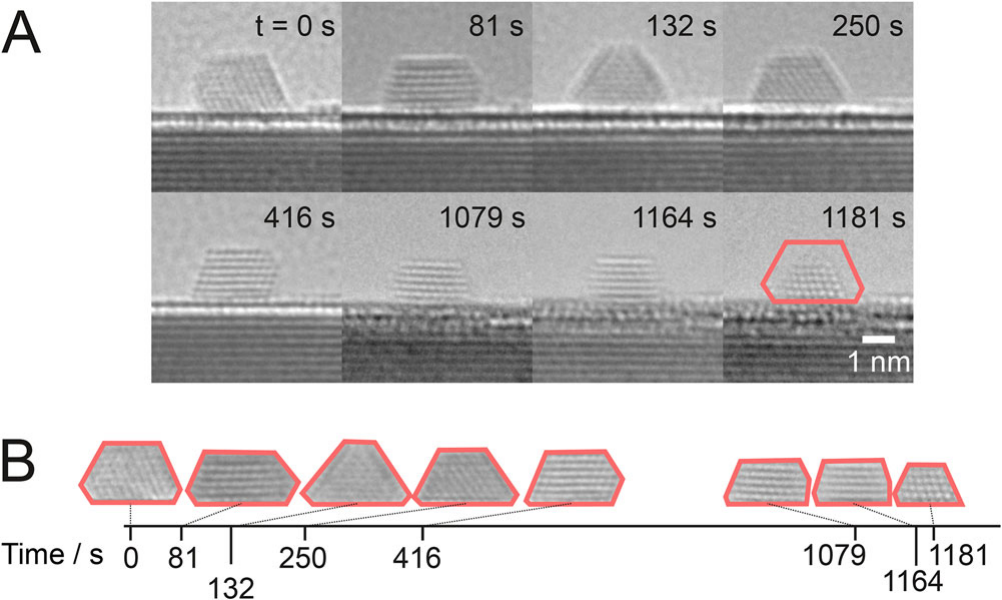
TEM images (A) of a Pt particle during redispersion (time range of 0 s to 1181 s) under oxidizing conditions at 500°C. At 1181 s, a red silhouette is added showing the initial Pt particle size at the time point 0 s. The cut‐out Pt particles and silhouettes (red line) at the respective time (B) show morphological changes as well as a qualitative decrease in particle size.

In the first step, a region of interest with CeO_2_ nanocubes oriented parallel to the electron beam was defined. After reduction and switching to oxidizing conditions (500°C, 1.5 mbar O_2_), a series of images was recorded over a time frame of 30 min. To limit the influence of the electron beam, the beam was blanked in between the measurements. Since periodic electron beam exposure could potentially influence the observed trends, the delay between image acquisitions was varied. Figure 2A shows 8 TEM images of a Pt particle over a time frame of ∼20 min. It can be observed that the shape of the particle is changing strongly under the applied conditions. Also, these morphological changes go hand in hand with an apparent loss in particle size. This variation is emphasized in Figure 2B, where the silhouettes of the particle are shown on a timescale from 0 s to 1181 s. The redispersion capability is governed by the metal–support interaction, which depends on the exposed CeO_2_ facets and thus on the particle morphology, as established in previous studies [51]. Therefore, the observations are well in line with previous studies, where the noble metal redispersion was linked to the formation of nano‐pockets on the {100} facet, the primary exposed facet for cube‐shaped CeO_2_ [44, 52]. Also, redispersion via adsorbed single Pt atoms is discussed, which would enable the redispersion process on, e.g., the {111} facet [53] and would also influence the interaction with the support in terms of Pt‐O‐Ce coordination number [54]. Since the {100} surface is not the thermodynamically preferred orientation under oxidizing conditions, the in situ restructuring of ceria surface was shown to occur that leads to step edges able to host Pt adatoms [55, 56].

**FIGURE 3:**
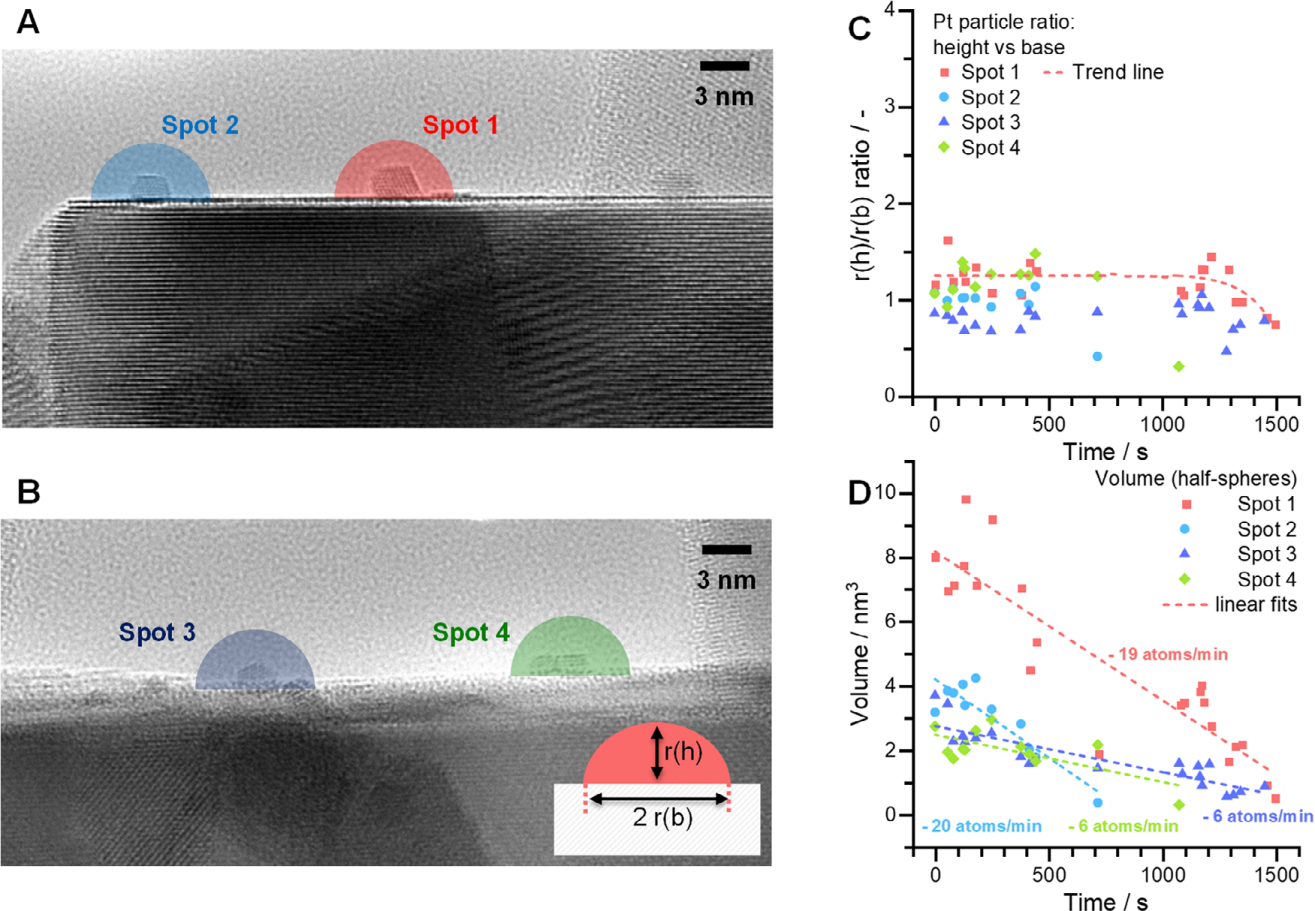
The TEM images (A, B) show the region of interest with four Pt NPs highlighted in red (spot 1), blue (spot 2), dark blue (spot 3), and green (spot 4). The schematic inset in B shows the definition of r(h) and r(b), the height and length of the base of the NPs, respectively. These were used to estimate the nanoparticle volume assuming half spheres. For the four spots the time‐dependent height‐to‐base ratio (C) and the rate of redispersion (D) are given at 500°C in 1.5 mbar O_2_.

An analogous behavior was observed for further noble metal particles in the region of interest. As depicted in Figure 3A,B, four Pt NPs could be identified in total, which were orthogonally oriented with respect to the electron beam (1 to 4 labelled spots; Figure S11 showing a fifth Pt NP, which is overlapped by an adjacent CeO_2_ cube). Assuming a hemispherical shape (cf. Figure S12—faceted reconstruction assuming y‐axis symmetry and the same particle length in x and y axis, illustrated for the NP at spot 1 at *t* = 0 min), the volume of Pt nanoparticles could be obtained as a function of time by measuring the particle height r(h) orthogonally to the CeO_2_ surface and base length r(b) with the aspect ratio being defined as r(h)/r(b). The variations of these parameters over time allow a direct quantification of the shrinking and redispersion of Pt NPs under oxidative conditions (cf. Figures S7–S9). The aspect ratio (r(h)/r(b)) is reported in Figure 3C. Accordingly, a value around 1 indicates a perfect hemisphere, while a value > 1 describes a more convex and a value < 1 a flatter morphology. However, the shape of the NPs is not half‐spherical but strongly facetted in the O_2_‐rich atmosphere (Figure 2A). Quantum effects dominate the Pt properties in this size regime (< 1.5 nm) [57, 58], limiting straight‐forward shape simulations via, e.g., Wulff or Winterbottom construction. Furthermore, the rapid change in the Pt NP shape illustrates that only low energy barriers need to be overcome between the different structures under these conditions. Precise shape determination is therefore not possible based on 2D TEM images. However, the trends in the obtained r(h)/r(b) values for hemispherical shapes of Pt NPs capture the relevant size changes.

Based on this analysis strategy, the loss in the particle volume (Figure 3D) and the morphological changes (Figure 3C) were estimated from the experimental aspect ratio parameters. In general, an enhanced stability of Pt single atoms, which presumably also increases the redispersion rate, is achieved on the {100} facet, which is claimed to stabilize the single Pt atoms in nanopockets [27, 44, 59]. In this study, all Pt NPs display an aspect ratio around 1 at the start of the experiment, which is maintained through the redispersion process. Hence, all particles seem to shrink proportionally. For all particles, but particularly visible for spot 1, the r(h)/r(b) ratio reaches values below 1 at the end of their lifetime. This behavior correlates with the flattening of the particles and higher contact area with the CeO_2_ support before the full redispersion occurs. In such a scenario, a single layer of Pt on CeO_2_ would be the final stage before atomic dispersion. Indeed, so‐called Pt *rafts* were already reported after mild reduction treatments on high surface area CeO_2_ [48]. Accordingly, it can be assumed that these noble metal ensembles represent a first stage during the NM agglomeration process and a last step for the full redispersion of Pt clusters.

While the analysis is based on a sample region exposing four nanoparticles, the findings nonetheless reveal consistent trends, which suggest that, irrespective of the initial particle volume (∼2.5 nm^3^ for spot 4, ∼8 nm^3^ for spot 1) the slope of the atoms loss, deduced from the decrease in Pt NP volume with time, can be described by a linear function (Figure 3D). However, the rate of the redispersion process seems to depend strongly on the surroundings of each Pt nanoparticle. Spots 1 and 2 in Figure 3 show the highest rate of redispersion, which could be estimated to approx. 19 to 20 Pt atom loss per minute. In both cases, Pt NPs were rather isolated on a plane CeO_2_ surface. Spots 3 and 4 show much lower redispersion rates of 6 Pt atoms per minute. In these cases (highlighted in Figure S10), the observed Pt particles were close to other Pt particles (Spot 3 and Spot 4) or to a crossing point of two adjacent CeO_2_ cubes (Figure S11, Spot 5). At 500°C, the noble metal redispersion process is assumed to occur more localized on CeO_2_ surface since gas phase PtO_2_ was observed mostly at temperatures of >650°C [36, 41]. Therefore, the maximum level of Pt redispersion seems to be limited by the concentration of Pt atoms per CeO_2_ particle. This behavior is in agreement with the previously reported beneficial effect of a higher surface noble metal concentration on the formation rate of noble metal clusters [27, 32]. Here, the opposite effect is demonstrated: a higher local concentration of Pt NPs leads to a lower redispersion rate. In situ microscopy on a larger scale, as demonstrated for Pt nanoparticles in zeolites [60], could serve as a complementary approach to assess the average particle lifetime in future studies.

### 2.3 Detachment Mechanism of Pt Atoms From NPs

**FIGURE 4:**
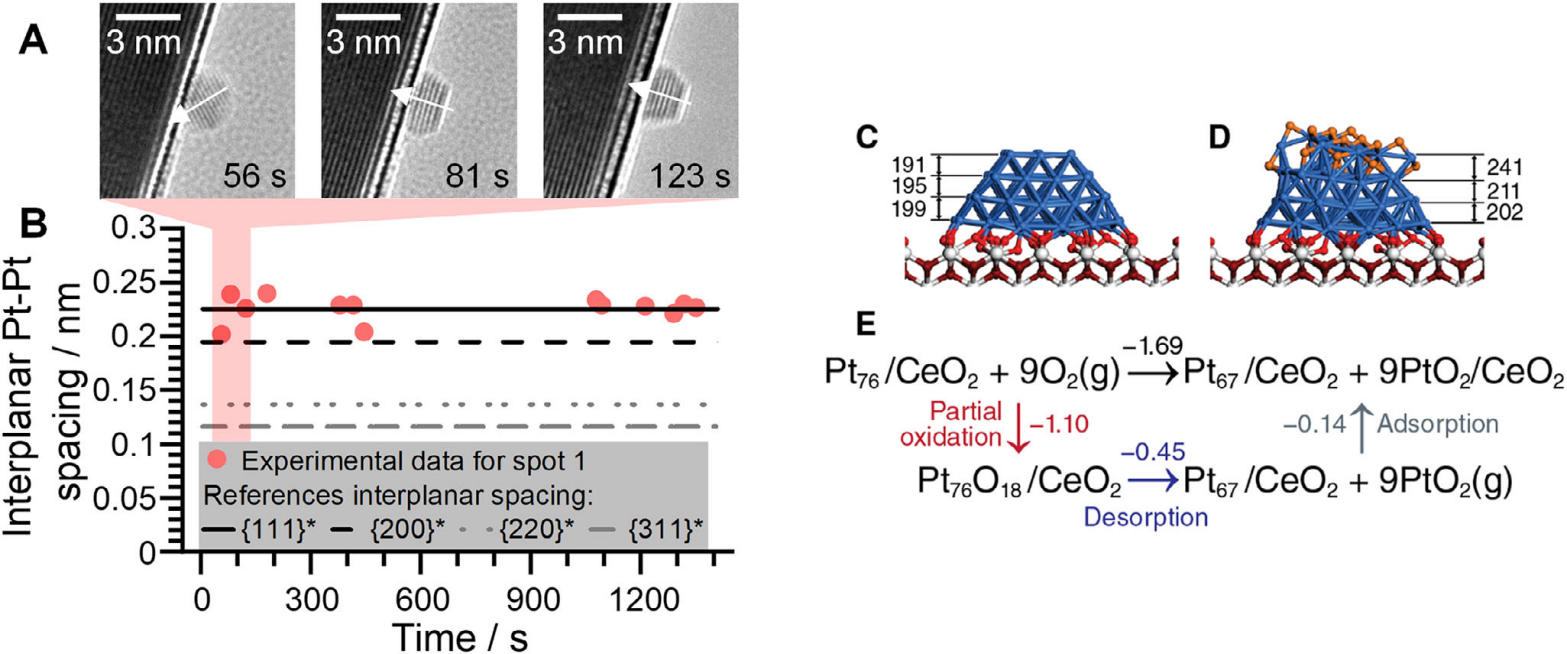
ETEM images during the redispersion process (A) under oxidizing conditions (500°C, 1.5 mbar O_2_; reference values from ICSD [62] 76153). Average interplanar Pt‐Pt spacing (B) of a Pt particle over time. DFT‐calculated structures of a Pt_76_ NP in a reduced (C) and partially oxidized (D) state at its surface supported on CeO_2_(100), showing an increase in the interplanar Pt‐Pt spacing during oxidation. (E) Deconvolution of the free energy of redispersion into partial oxidation of the NP (red), desorption of PtO_2_ molecules (blue), and adsorption of PtO_2_ on ceria (gray). Energies in eV per PtO_2_ molecule. Distances in pm in (C) and (D).

Besides the absolute loss in atoms or in volume per time, the recorded TEM images provide information on the redispersion mechanism. As indicated by the performed experiments and in line with previous literature reports [33], temperatures above 400°C and oxidizing conditions trigger the redispersion of Pt particles. This process may start with the full oxidation of the NP, followed by the detachment of atoms. Hereby, the interaction with the reducible support can act as a driving force for oxygen spillover and thus oxidation of the noble metal, as recently demonstrated for Ru/TiO_2_ [61]. This hypothesis can be verified by the measurement of the average interplanar Pt‐Pt distance, which can be extracted as function of time (Figure 4A,B). As mentioned above, the orientation of the Pt lattice was mostly parallel to the surface of the CeO_2_ support, and the observed Pt‐Pt lattice distance was found to be between 0.20 and 0.25 nm. In particular, distances around 0.227 nm are close to the Pt‐Pt distance in the {111} planes of metallic Pt (0.2265 nm based on a *fcc* lattice constant of 0.39231 nm, reference values from ICSD [62] 76153). At two time points during the redispersion process, an average interplanar distance of 0.196 nm was measured, which can be assigned to the {200} planes of metallic Pt. Considering that the interplanar distance of a fully oxidized PtO_2_ NP would be significantly larger (average distance of 0.399 nm), it seems that at least the core of the Pt NPs stayed mostly reduced under the applied conditions (500°C, 1.5 mbar O_2_).

However, the interplanar distance in NPs is expected to be lower than in the bulk phase due to the interaction with the support. This is illustrated by the DFT calculations of a 4‐layer Pt NP supported on the CeO_2_ (100) plane (Pt_76_, Figure 4C), where a 1% contraction of the average interplanar distance was computed. Nonetheless, at most time points during the NM redispersion process, the interplanar distances observed by TEM were larger than the bulk value. This expansion of the lattice structure cannot be attributed to imaging artifacts or misalignment, as the CeO_2_ lattice, which was used as a reference, remained constant throughout the sample treatment (Figure S6). Thus, the origin of expansion can be traced back to the partial oxidation of the Pt NPs. While the interplanar distance of a fully oxidized PtO_2_ NPs would be significantly larger (0.399 nm), partial oxidation of a single layer is expected to result in a moderate expansion of the average lattice distances, potentially explaining the experimental observations. For instance, in our DFT model, the oxidation of the top layer (Pt_76_O_18_, Figure 4D) results in an increase in the average interplanar distance from 0.195 nm to 0.218 nm. This implies that the larger the NP, the smaller the expected impact of single‐layer oxidation on the average interlayer distance.

Thus, the slight interlayer expansion allows us to postulate a redispersion mechanism involving the partial oxidation of Pt NPs. Considering that the used DFT model for the supported Pt NP is rather small compared with the experimentally measured ones and has an aspect ratio r(h)/r(b) below 1 (Pt_76_, r(h)∼0.6 nm, r(b)∼0.7 nm) corresponding to a particle at the end of its lifetime, we assumed a layer‐by‐layer oxidation and detachment rather than a proportional oxidation and shrinkage of the entire Pt NP. In this case, the redispersion may operate via the ceria‐assisted detachment of these oxidized PtO_x_ layers, which subsequently adsorb on the ceria surface as isolated species. Thanks to DFT modeling, the free energy Δ*G* of the supported Pt_76_ redispersion on ceria was computed by considering the thermodynamic equilibrium at 500°C in 1.5 mbar of O_2_ and 1 x 10^−8^ mbar of PtO_2_ in gas phase for the calculation of the respective chemical potentials, as shown in Figure 4E. The whole reaction process is largely exergonic, considering the redispersion of Pt_76_ into Pt_67_ and PtO_2_ surface species (−1.69 eV/PtO_2_). Such reaction can be decomposed into three other processes. First, the oxidation of the terminal layers of Pt_76_ is exergonic (−1.10 eV /PtO_2_). This step is followed by a simultaneous exergonic desorption of PtO_2_ (−0.45 eV/PtO_2_) and further adsorption on ceria (−0.14 eV/ PtO_2_). An alternative pathway is the diffusion of PtO_2_ from the top layer to the triple phase boundary, a process that is also exergonic (−2.98 eV, see Figure S14), followed by the diffusion from the triple phase boundary to the ceria surface, a process that cannot be easily investigated using our periodic model due to size limitations. In short, the redispersion through partial oxidation of Pt NP surface/perimeter sites is thermodynamically favorable step‐by‐step according to the DFT modeling. Nevertheless, to rigorously validate the proposed mechanism, further studies are required, particularly with higher temporal resolution, since in the second regime, the structural changes were at times too rapid to be fully resolved by the microscope. Such measurements, together with complementary spectroscopy and DFT‐calculations, would enable a more detailed elucidation of the detachment mechanism.

### 2.4 Influence of the Atomic Redispersion Kinetics on the Integral Oxidation Activity

**FIGURE 5:**
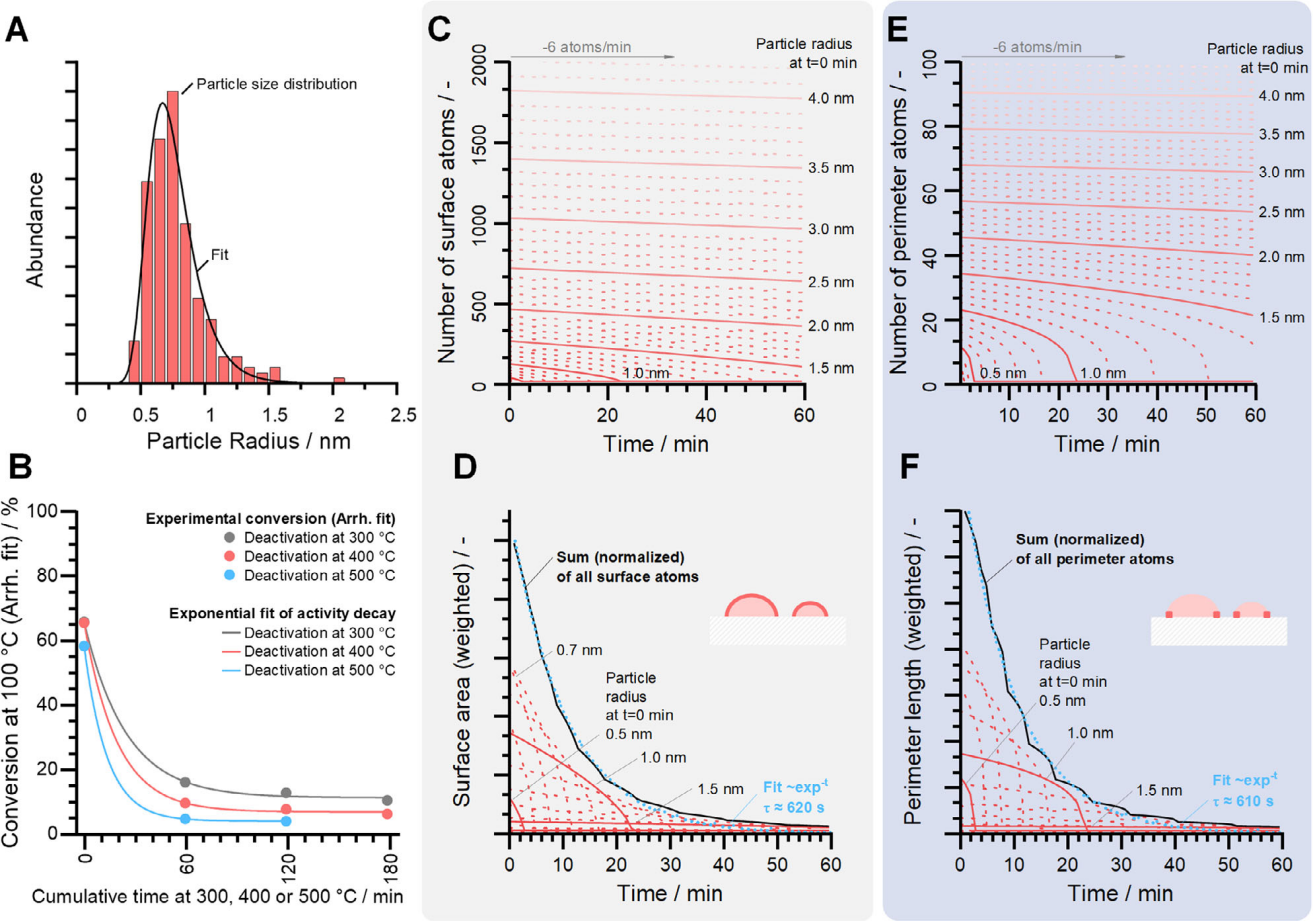
Pt particle size distribution after a reductive treatment with corresponding Gumbel fit (A). Catalytic activity in terms of CO conversion at 100°C (B) for three consecutive temperature programmed activity tests to 300°C, 400°C, and 500°C, respectively (see Figure S4). In between each light‐off, the catalyst was kept at the highest temperature for 60 min. To enhance the reliability of the experimental data, an Arrhenius fit was first performed, and based on this, the conversion was extrapolated. Number of Pt surface atoms (C) and perimeter atoms (E) over time for different half‐spherical NPs up to a radius of 5 nm. Weighted number of surface atoms (D) and perimeter atoms (F) obtained by the multiplication of the number of surface atoms as a function of the particle size by the fit of the particle size distribution.

Previous studies have shown [34, 63] that the oxidative redispersion of Pt NPs in ceria‐supported catalysts into highly dispersed Pt oxide species or single atoms like Pt^2+^ species on CeO_2_ is responsible for the gradual loss of active sites. Accordingly, the evolution of the overall activity and the catalyst deactivation profile should be reflected by the noble metal redispersion rate. Based on the ETEM observations of a constant atomic detachment rate, the overall rate of redispersion at different temperatures was estimated. In all cases, a constant rate was applied for the Pt atoms detaching from a given NP (Figure 5C, assumed to be constant with a loss of 6 atoms per minute, the minimum redispersion rate experimentally observed at 500°C in the ETEM) to the initial NP size distribution measured for Pt/CeO_2_ (Figure 5A, fit using a Gumbel size distribution). The resulting time evolution of the total number of active surface atoms (considering dispersed Pt atoms as inactive) in the sample is represented in Figure 5D.

For the catalyst used in this study, the majority of Pt NPs have an initial radius of up to 1 nm, which is calculated to fully redisperse within the first 20–30 min at 500°C (Figure 5D,F). After this time, only the larger NPs are expected to contribute to the total amount of surface atoms despite their low initial share. The sum of all surface atoms (black line in Figure 5D) seems to follow an exponential decay, which is the result of the superimposition of the evolving particle size distribution due to a linear loss of Pt atoms. The exponential time constant τ_redisp_ of the redispersion process at 500°C was determined to be ∼620 s. An analogous calculation conducted for the number of perimeter sites, which were reported to be the active species for reduced Pt NPs on CeO_2_ [64], led to a comparable time constant of ∼610 s (Figure 5E,F) and slightly improved the exponential fit at time points larger than 20 min. These trends further emphasize that the interfacial sites between the noble metal particles and the CeO_2_ support are crucial for the catalytic performance of these catalysts, in line with previous studies [23, 65].

While a direct correlation of the ETEM results and the catalytic data is challenging due to the pressure gap of 3 orders of magnitude, one can still compare the trends derived from these experiments. To characterize the stability of Pt NPs that is mirrored in the evolution of the catalytic activity, three consecutive CO oxidation light‐off/light‐out cycles (linear heating/cooling in reaction mixture) were conducted between 50–300°C in an oxygen‐rich gas mixture (1000 ppm CO, 8 % O_2_, N_2_). An initial Pt state comparable to that present in the ETEM experiments was achieved by the pre‐reduction of the Pt/CeO_2_ catalyst in 2% H_2_ at 300°C for 30 min prior to the catalytic tests. Between the light‐offs and light‐outs (Figure S4), the catalyst was held at 300°C for 60 min in reaction mixture. The same experimental procedure was repeated for two additional holding temperatures of 400°C and 500°C. The CO conversion at 100°C was calculated based on an Arrhenius fit of the experimental data and is reported in Figure 5B as a function of catalyst holding cumulative time at 300°C, 400°C or 500°C, respectively. Overall, the following applies: the higher the exposure temperature during the light‐off/light‐out cycles, the faster the loss in the CO oxidation activity. Furthermore, in all cases, the strongest deactivation was observed between the first and second light‐off (0 h and 1 h holding time at 300°C, 400°C, or 500°C). This behavior of pre‐reduced Pt/CeO_2_ catalysts is in agreement with previous studies on NM redispersion in oxidative atmospheres as a function of temperature [26, 65]. In addition, the time‐dependent loss in activity measured in this study could be fit using an exponential decay function—analogously to the above‐mentioned Pt redispersion process. The exponential time constant τ_cat_ of the catalytic activity was found to be ∼1500 s for the deactivation at 300°C, ∼1200 s at 400°C, and ∼800 s at 500°C. Under the assumption that these time constants are inversely proportional to the redispersion rate, it is also possible to estimate the activation energy for the Pt redispersion of about 11 kJ/mol using the Arrhenius equation (Figure S13). Considering the different partial pressures in the reaction mixtures, the similarity of τ_cat_ and τ_redisp_ at 500°C is remarkable (∼800 s vs. ∼600 s). Moreover, since these time constants were obtained at very different O_2_ partial pressures, this suggests that oxygen adsorption on Pt surface is not the rate‐limiting step during catalyst deactivation. Instead, the dominant factor is likely the kinetic barrier associated with the detachment of a PtO_x_ unit from the perimeter sites, what is in line with the Hammond principle based on the DFT computed change in exergonicity: it is greater for the surface oxidation of the Pt NP (‐1.1 eV/PtO_2_) than for the detachment of PtO_2_ species (−0.45 eV/PtO_2_, Figure 4E). However, this hypothesis requires further investigations and should be addressed in more detail in future studies, e.g., using different temperatures, different ceria facets, or surface noble metal loading.

The difference of 200 s in τ between the ETEM and catalytic activity tests, which translates to roughly 25 % of the absolute values, could be the result of either a systematic underestimation of the particle size distribution, local temperature differences due to the introduction of cooled gas during the in situ TEM experiments, or the presence of CO in the catalytic tests. As reported in our earlier studies, CO leads to the formation of Pt clusters under reaction conditions and counteracts the “redispersion potential” of O_2_ or could also lead to the formation of NPs during cooling in reaction mixture [27, 30, 31]. Consequently, its presence seems to stabilize Pt clusters and nanoparticles by hindering dispersion despite the higher O_2_ partial pressure in the system. This could also explain the lower threshold of the activity in the exponential fit, which ranges from 11 % conversion (deactivation at 300°C) to 3 % (deactivation at 500°C) and does not approach 0 %. Whether other adsorbates, such as water or hydrocarbons, accelerate or hinder the redispersion is not clear yet, based on literature reports. Nie et al., claimed a higher activity and long‐term stability for Pt/CeO_2_ single atom catalyst in the presence of hydroxyl groups on the surface [66]. In their study, Wu et al., investigated the influence of water and came to the results that the presence of H_2_O hinders the redispersion of Pt on cube‐shaped CeO_2_ under alternating H_2_ and O_2_ feed [37]. According to a recent study by Li and coworkers, NO promotes redispersion, particularly for highly coordinated Pt species at low temperatures of 200°C, while stabilizing low‐coordinated ones [39]. Varying the chemical potential and temperature could represent a way to effectively increase the lifetime of the NPs.

Overall, the time‐dependent diminishment of the catalytic activity appears to follow a similar logic to that observed in the loss of Pt surface sites due to redispersion. While the latter model is relatively simple and does not consider how the reaction rates would vary depending on the type of Pt surface atoms (perimeter sites, kink or edge sites), this atomic picture can be used as a first approximation to elucidate the primary mechanisms underlying the macroscopically observed catalytic deactivation. On the other hand, the catalytic measurements also imply that the time constant of catalytic deactivation is directly proportional to the temperature, with approx. −3.4 s/K slope according to a simple linear regression (Figure S5). The temperature range for rate‐prediction is limited since on the one hand gas‐phase migration [67] sets in at around 800°C and segregation phenomena [43] can occur. A significant reduction in support surface area can induce an increase in surface noble metal concentration and promote Pt autoreduction at higher temperatures. Despite these effects, the results remain applicable for predicting the evolution of catalytic activity across the investigated temperature range. Conversely, assuming that the main reason for deactivation under the given conditions is in fact oxidative redispersion, it also implies that the dispersion kinetics are linearly dependent on the temperature. This outcome allows to make structural predictions on the NM particle size distribution and opens the possibility to tune the ratio between metal NPs and highly dispersed entities by carefully controlled oxidative treatments followed by a mild reductive treatment. This could be a straightforward approach to synthesizing bifunctional catalysts for specific applications.

## 3 Conclusion

This study presents the quantification of the kinetics of atomic‐scale structural transformation of nanoparticles using the case of noble metal redispersion on a defined CeO_2_ surface, which could be correlated with the evolution of the CO oxidation activity of a Pt/CeO_2_ catalyst as well as its deactivation pattern in a conventional fixed‐bed reactor. Systematic ETEM experiments uncovered that the volume of the noble metal particles decreases linearly in an oxidative gas atmosphere. However, the Pt redispersion rate is strongly influenced by the local environment (e.g., neighboring Pt particles). A simplified mathematical model was used to predict the time‐dependent evolution of surface atoms and link it to the experimentally observed catalytic activity. Both the catalytic decay and the redispersion process followed exponential trends at 500 °C, with comparable time constants of approximately 800 and 600 s, respectively. Additionally, DFT calculations were performed to investigate the stability of the supported reduced and partially oxidized Pt NPs on CeO_2_(100) at the end of their lifetime, shortly before full redispersion. The comparative analysis of the experimental and theoretical results showed that the morphological changes of the catalyst are connected to a partial reoxidation of the noble metal surface sites, while a bulk NP oxidation was not observed under the given conditions. Furthermore, a linear relationship between catalytic activity loss and deactivation temperature was observed, suggesting a temperature‐dependent redispersion mechanism. These findings mark a significant step toward a predictive control of the structural and catalytic behavior in Pt/CeO_2_ nanomaterials under operating conditions.

More broadly, this work demonstrates that key processes such as redispersion and morphological nanoparticle restructuring—often assumed to occur over extended timescales—can unfold within seconds to minutes. This highlights the critical importance of time‐resolved in situ techniques, such as electron microscopy and operando X‐ray methods, in capturing the dynamic nature of functional materials. Understanding these rapid structural transformations is essential for the rational design of catalysts with improved stability and performance under realistic operating conditions. Next steps are to confirm the temperature dependence, the environmental impact (e.g., different O_2_‐partial pressure), and noble metal type on the deactivation kinetics for further catalysts tending to redispersion/particle formation, composite formation, or morphological changes.

## Author Contributions

The manuscript and supporting information were written through contributions of all authors. A.S., D.L., and C.M. performed the DFT calculations. Sample preparation and treatment were conducted by F.M., A.B., S.L. and P.D. Catalytic testing was done by A.B. and F.M., XAS measurements were performed and evaluated by F.M., ETEM experiments by M.A., T.E., and F.M. F.M., M.C., P.V., C.M. and J.D.G. were responsible for the concept, experimental design, and arrangement of the paper. All authors have given approval to the final version of the manuscript.

## Funding

Financial support was provided by ANR (DYCAT project, Grant No. ANR‐19‐CE05‐0038) and DFG (DYCAT project, Grant No. 431423888 and SFB 1441 TrackAct, Project‐ID 426888090).

## Conflicts of Interest

The authors declare no conflicts of interest.

## Supporting information



The Supporting Information contains the experimental and methods section, further catalytic and characterization data, including x‐ray absorption spectroscopic and electron imaging data. Additionally, a movie is provided showing ETEM images during the redispersion of Pt particles. This material is available free of charge via the Internet at springer.com. **Supporting File 1**: anie73590‐sup‐0001‐SuppMat.pdf.

**Supporting File 2**: anie73590‐sup‐0002‐MovieS1.mp4.

## Data Availability

The data that support the findings of this study are available in KITopen at https://doi.org/10.35097/xjsgtra0810qf5g2.
