# Supplementary material for: Predicting Catalyst Performance From Pt/CeO2 Redispersion Kinetics
Source: Angew Chem Int Ed Engl. 2026 Jul 26;65(39):e4743891. doi: 10.1002/anie.4743891 (PMC13592305; doi:10.1002/anie.4743891)
Supplement: Supplementary file 1 [file ANIE-65-e4743891-s001.pdf]

Supplementary Information: Predicting Catalyst Performance from Pt/CeO_2 Redispersion Kinetics

Florian Maurer[†], Agustin Salcedo[‡], Maria Casapu[†], David Loffreda[‡], Arik Beck[†], Paolo Dolcet[†,&], Mimoun Aouine[§,«], Thierry Epicier[§,«], Stephane Loridant[§,«], Philippe Vernoux[§,«], Carine Michel[‡,*], Jan-Dierk Grunwaldt[†,#,*]

† Institute for Chemical Technology and Polymer Chemistry (ITCP), Karlsruhe Institute of Technology (KIT), Engesserstraße 20, 76131 Karlsruhe, Germany

‡ CNRS, ENS de Lyon, Université Claude Bernard Lyon 1, LCH, UMR 5182, 69342 Lyon Cedex 07, France

& Department of Chemical Sciences, University of Padova, Padova 35131, Italy

§ Université Claude Bernard Lyon 1, CNRS, IRCELYON, UMR5256, Villeurbanne, F-69100.

« Matériaux, Ingénierie et Science, UMR 5510, CNRS, INSA de Lyon, Université de Lyon, 2 Avenue Albert Einstein, 69626 Villeurbanne, France

Institute of Catalysis Research and Technology (IKFT), Karlsruhe Institute of Technology (KIT), Hermann-von-Helmholtz-Platz 1, 76344 Eggenstein-Leopoldshafen, Germany

* Jan-Dierk Grunwaldt, Institute for Chemical Technology and Polymer Chemistry (ITCP), Karlsruhe Institute of Technology (KIT), Engesserstraße 20, 76131 Karlsruhe, Germany

Institute of Catalysis Research and Technology (IKFT), Karlsruhe Institute of Technology (KIT), Hermann-von-Helmholtz-Platz 1, 76344 Eggenstein-Leopoldshafen, Germany

Email: grunwaldt@kit.edu

* Carine Michel, Ecole Normale Supérieure de Lyon, CNRS, Laboratoire de Chimie UMR 5182, 46 allée d'Italie, F-69364 Lyon, France

Email: carine.michel@ens-lyon.fr

Table of Contents

Materials and Methods S2
- Section S1: Synthesis of cubic CeO_2 .... S2
- Section S2: Pt impregnation S2
- Section S3: *Ex situ* characterization S2
- Section S4: *In situ* X-ray Absorption Spectroscopy (XAS) S3
- Section S5: Environmental Transmission Electron Microscopy (ETEM) S3
- Section S6: Catalytic CO oxidation tests S4
- Section S7: DFT calculations S5

Supplementary Figures S7

References S17

Materials and Methods

Section S1: Synthesis of cubic CeO_2

Cube-shaped CeO_2 was synthesized according to the route developed by Mai *et al.*[1] For the synthesis, autoclaves with a volume of 1,220 mL (BR-1000, Berghof, T_{max} = 300 °C, p_{max} = 200 bar), an integrated stirrer (RV-100, Berghof) and a thermostatic jacket (BTM, Berghof) with a Teflon inlay were used. 120.0 g of NaOH (VWR Chemicals) and 10.86 g of $Ce(NO_3)_3 \cdot 6\ H_2O$ (99%, Sigma-Aldrich) were dissolved in 425 mL and 75 mL of deionized H_2O, respectively. Then, the Ce-precursor solution was slowly added to the NaOH containing solution under constant stirring. The closed autoclave was then heated to 167 °C. The resulting pressure in the autoclave was approx. 6 bar. The separated solid was washed with deionized water and dried for 12 h at 70 °C.

Section S2: Pt impregnation

1 wt.% of Pt was loaded onto the CeO_2 cubes by incipient wetness impregnation. For this purpose, $(NH_3)_4Pt(NO_3)_2$ (99%, Alfa Aesar) was dissolved in purified water (purified water, ROTIPURAN). The obtained aqueous solution was added dropwise to the CeO_2 powder, which was constantly grinded in a mortar. Finally, the catalyst was calcined at 300 °C in static air.

Section S3: *Ex situ* characterization

N_2 physisorption was applied to determine the surface area of the cubic CeO_2 support before impregnation with Pt. The multipoint measurement was performed at −196 °C according to the Brunauer-Emmett-Teller-method (BET)[2] using a BELSORP-mini instrument (MicrotracBEL).
The as-prepared CeO_2 nano cubes were characterized by high-angle annular dark-field scanning transmission electron microscopy (HAADF-STEM) in a FEI Tecnai Osiris. The Pt particle size statistics of the specimens (counting >100 particles) were determined on HAADF-STEM images by using the Fiji software[3].
The weight loading of Pt was determined *via* inductively coupled plasma optical emission spectroscopy (ICP-OES). The measurements were performed at the Institute of Applied Materials at KIT using a 720/725 emission spectrometer (Agilent).

Section S4: *In situ* X-ray Absorption Spectroscopy (XAS)

Beamline: XAS measurements in fluorescence mode were performed at the SuperXAS Beamline[4,5] at the Swiss Light Source (SLS). A dual channel monochromator with Si(111) crystals were used to tune the incident photon energy to the Pt L_3 edge. A 1-element Si drift detector was used to scan the intensity of emitted photons.

In situ setup: A quartz capillary reactor[6] (outer diameter: 1.5 mm; inner diameter: 1.48 mm) heated by a hot air gas blower was used for the tests. For the *in situ* measurements, gases were dosed using mass flow controllers (MFC, Bronkhorst). The product feed consisting of 8 % O_2 in He for the pretreatment and 2 % H_2 in He for the temperature programmed reduction was monitored at the outlet of the reactor using a mass spectrometer.

Procedure: 16 mg Pt/CeO_2 catalyst (pressed and sieved to 100–200 µm) was loaded into the quartz capillary reactor and fixed using quartz wool plugs. First, the surface of the nano material was cleaned by a temperature programmed oxidation (8% O_2 in He), for which the catalyst was heated to 400 °C with 10 K/min. After cooling down, the gas mixture was changed to reducing conditions (2 % H_2 in He) and the temperature programmed reduction was started heating the catalyst to 300 °C with 3 K/min. By recording one X-ray absorption near edge structure (XANES) spectrum every 7-8 minutes, a temperature resolution of ~30 K was achieved.

Data Treatment: The XAS spectra were evaluated using the Athena application of the Demeter software package[7] (version 0.9.21). For the linear combination fitting, two references were chosen: Pt/CeO_2 after oxidation in 8 % O_2 at 400 °C and Pt/CeO_2 after reduction in 2 % H_2 at 300 °C.

Section S5: Environmental Transmission Electron Microscopy (ETEM)

Instrument and setup: Environmental Transmission Electron Microscopy measurements were conducted in an image Cs aberration corrected FEI-Titan ETEM (80-300 kV). The Pt/CeO_2 material was then transferred on heating chips (Wildfire, DENSsolutions). Prior to the loading of the sample, the holder was plasma cleaned. Gases were dosed by mass flow controllers (Brooks) and premixed before entering the microscope. During the experiment, the pressure was monitored close to the sample and was set to 19 mbar H_2 for the reduction, 17-18 mbar O_2 for cleaning and 1.5 mbar for the redispersion experiments. To minimize beam effects and keep in general the

electron dose as low as possible during the experiments, the electron beam was blanked for most of the pre-treatments (except confirmation of particle formation and control of position drift) and during the redispersion experiment, images were only recorded for few seconds at the given time points. The time between image acquisition was varied to further rule out trends due to regular exposition to the electron beam.

Procedure: First, the catalyst was oxidatively treated to remove possible carbon containing residuals (400 °C in 18 mbar O_2). Afterwards, the sample was cooled to room temperature and the pressure was set to ultra-high vacuum (UHV) conditions. Then, H_2 was introduced until a pressure of 19 mbar was achieved at the sample. The catalyst was then heated to 500 °C for 105 min. Subsequently, HAADF images were recorded to confirm Pt nanoparticle formation. For these images, the pressure and temperature were reduced (6 mbar H_2 and 300 °C) to decrease the influence of the electron beam. At 300 °C, UHV conditions were set before switching to 1.5 mbar of O_2. The catalyst was then heated stepwise to 500 °C, while controlling the sample position at selected regions of interest. The sample was held for ~40 min at 500 °C under oxidizing conditions until most particles in the region of interest fully redispersed. The particle size (distribution) and lattice parameters were evaluated using the FIJI software[3].

Section S6: Catalytic CO oxidation tests

Setup: A quartz plug-flow reactor (outer diameter: 10 mm; inner diameter: 8 mm) was used to evaluate the catalytic performance. The reactor itself was placed inside a conductively heated furnace and the temperature during the reaction was controlled using two thermocouples, which were located in the reactor, up- and down-stream of the catalyst bed (approx. at 1 cm distance). Gases were dosed using MFCs (Bronkhorst) which were mixed before the reactor in heated (120 °C) stainless steel tubings. The concentration of the product stream was quantified at the outlet of the reactor by means of a Fourier-transform infrared spectrometer (FTIR, MultiGas 2030, MKS Instruments). The reactor was bypassed to check the concentration of gases without conversion before and after the measurements.

Procedure: For the catalyst testing, 100 mg of 1 wt.% Pt/CeO_2 (pressed and sieved to a grain size of 125–250 µm) was diluted with 900 mg SiO_2 (sieve fraction 125–250 µm) and placed in the reactor. Quartz wool plugs were used to fix the catalyst bed during the testing. The CO oxidation reaction mixture contained 1000 ppm of CO, 8% O_2,

balanced in N_2. The total gas flow was set to 1 L/min resulting in a weight hourly space velocity (WHSV) of 60 000 L/g_{Pt}/h. The overall procedure consisted to multiple light-off/light-out cycles between ~50 °C and 300 °C, 400 °C or 500 °C (cf. Fig. S4C). Prior to each test series, catalyst pre-treatments in a reductive atmosphere (2 % H_2 in N_2) were conducted at 300 °C to form small, reduced nanoparticles. Since the signal-to-noise ratio was low for conversions < 5 %, an Arrhenius fit was performed for all light-off curves. Based on these, the conversions at 100 °C were calculated, which are depicted at in Fig. S4B.

Section S7: DFT calculations

Density functional theory (DFT) calculations were performed using VASP 6.2.1.[8,9] The exchange-correlation energy was calculated within the generalized gradient approximation (GGA), with the Perdew-Burke-Ernzerhof (PBE) functional.[10] Long-range dispersion corrections were implemented with the DFT-D3(BJ) approach.[11,12] The valence electrons were described in a plane-wave basis set with an energy cutoff of 400 eV, whereas the core electrons were represented with the projector-augmented wave (PAW) method.[13,14] Total electronic energies were calculated with a numeric precision of 10^{-6} eV. Dudarev's DFT+U scheme[15] was chosen to consider strong electronic correlation effects due to charge localization, and to compensate for the self-interaction error, with U_{eff} of Ce(4f) set to 4.5 eV. The residual forces hold on the nucleis were minimized to ±0.02 eV/Å.

The CeO_2(100) surface was modeled by developing a stoichiometric and neutralized slab, which is stepped (see for instance Ref.[16]), using a (6×6) supercell generated from the ceria bulk with an equilibrium lattice parameter of 5.455 Å at the PBE-D3(BJ)+U level, and with a vacuum layer of 27 Å. This ensured a sufficiently long distance between periodically equivalent adsorbed Pt nanoparticles preventing undesired interactions between them. A smaller (2×2) CeO_2(100) supercell was used to evaluate the adsorption properties of PtO_2 species. The Brillouin zone was sampled with a (3×3×1) k-point mesh for the smaller (2×2) cell, and only at the Γ point for the larger (6×6) cell.

For the calculation of the reaction free energies of Figure 4, we considered the free energy of reactants and products as equal to the total DFT energy, except for the gas phase O_2 and PtO_2 molecules, for which we used statistical thermodynamics to predict

their free energy (i.e. chemical potential). Under this approach, the chemical potential of a linear ideal gas molecule at a given temperature and $P°$ = 1 bar is calculated as:

$$\mu = E_{\mathrm{DFT}} + ZPE + U_{\mathrm{trans}} + U_{\mathrm{rot}} + U_{\mathrm{vib}} + k_B T - T(S_{\mathrm{trans}} + S_{\mathrm{rot}} + S_{\mathrm{vib}})$$

Were E_{DFT} is the total DFT energy, ZPE is the zero-point energy, and the remaining terms are the rotational, translational and vibrational energy and entropy contributions. The total DFT energy of O_2 was corrected to account for the overbinding predicted by GGA functionals.[17] More details on these chemical potential calculations can be found in a previous work.[18]

The chemical potential at partial pressures different from $P°$ is obtained as follows:

$$\mu(T,P) = \mu(T,P°) + k_B T \ln\left(\frac{P}{P°}\right)$$

We considered partial pressures of $P_{\mathrm{O_2}} = 1.5\,\mathrm{mbar}$ and $P_{\mathrm{PtO_2}} = 1.10^{-8}\,\mathrm{mbar}$, resulting in chemical potentials at 500 °C of −10.84 eV and −18.73 eV, respectively. The energies of adsorbed Pt_xO_y species are reported in Table S1.

Table S1. Total energy (direct output of DFT calculations) of Pt_xO_y species adsorbed $CeO_2(100)$. Total energies of the ceria surface are also provided.

System	Total DFT energy (eV)
$PtO_2/CeO_2(100)$	−409.10
$Pt_{67}/CeO_2(100)$	−2091.76
$Pt_{76}/CeO_2(100)$	−2148.85
$Pt_{76}O_{18}/CeO_2(100)$	−2256.28
$CeO_2(100)$ (2×2, 9L)	−390.23
$CeO_2(100)$ (6×6, 5L)	−1697.96

Supplementary Figures

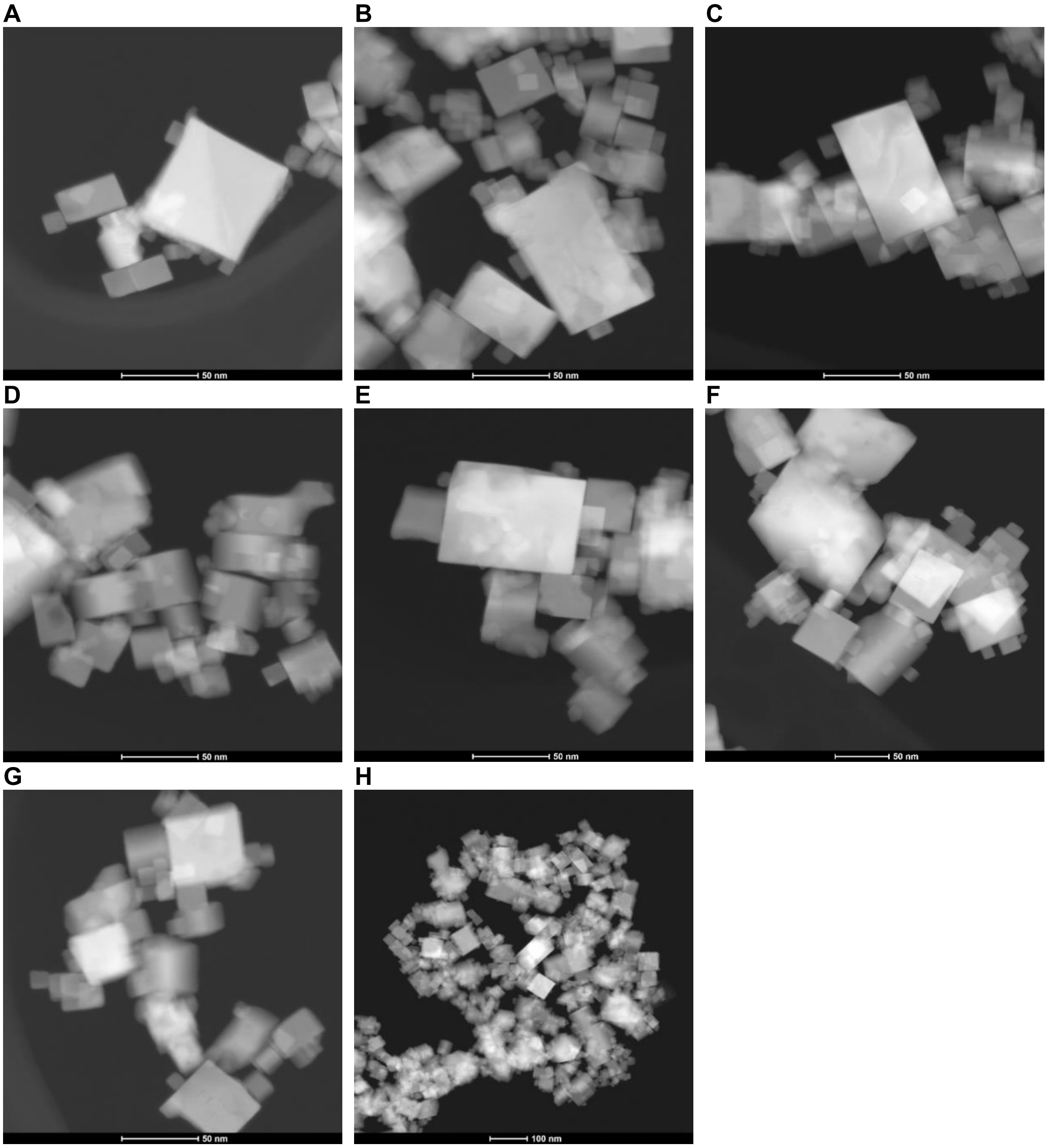

Figure S1. *Ex situ* HAADF-STEM images (A-D) of the as-prepared and (E-H) of the Pt-impregnated CeO_2 cubic nanoparticles.

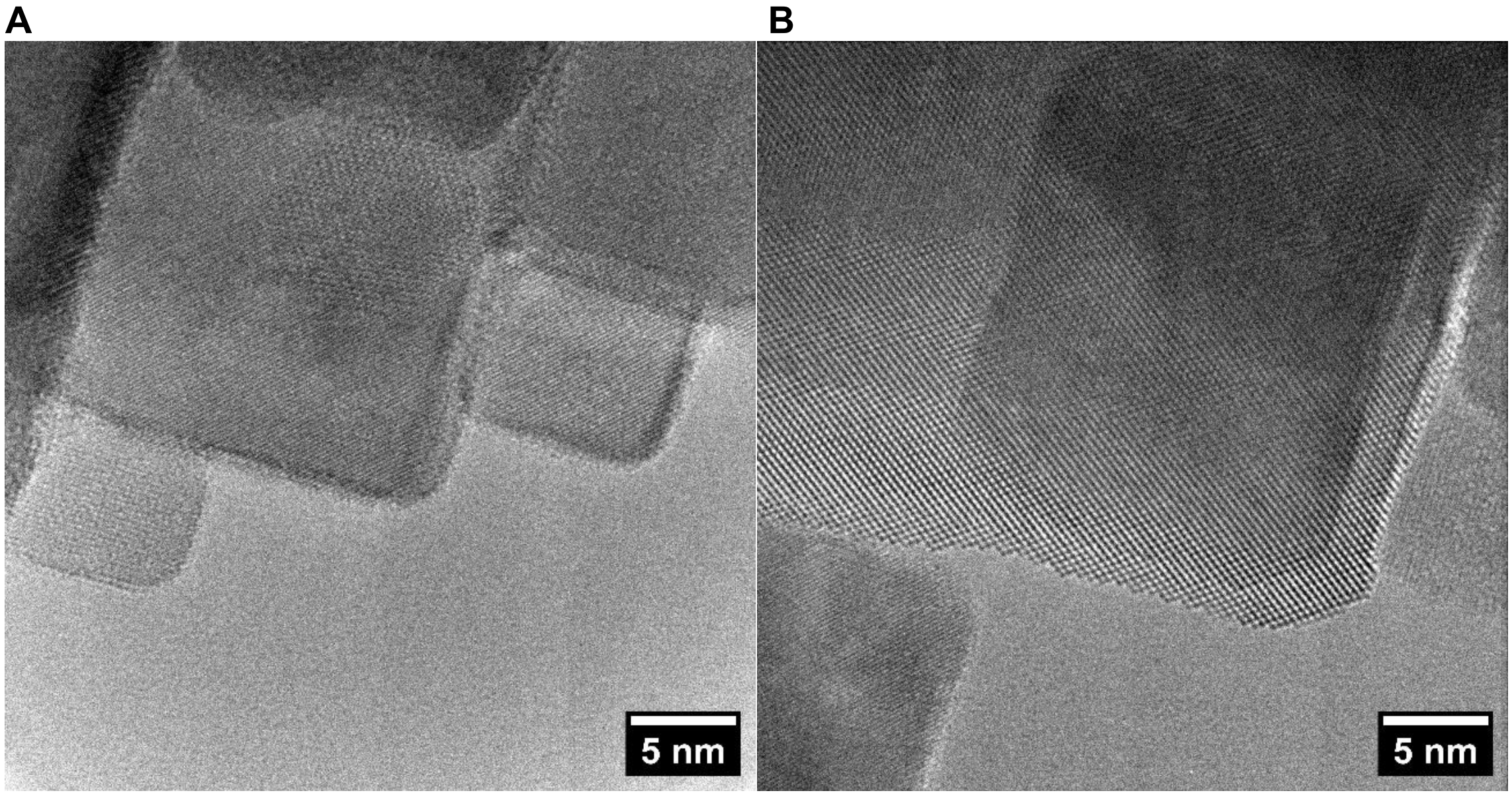

Figure S2. STEM images of the CeO_2 nanocubes after impregnation with Pt and consecutive calcination at 300 °C in air (1 bar). Prior to the acquisition, the surface of the nanoparticles was cleaned in the ETEM in 18 mbar O_2 at 500 °C for 30 min.

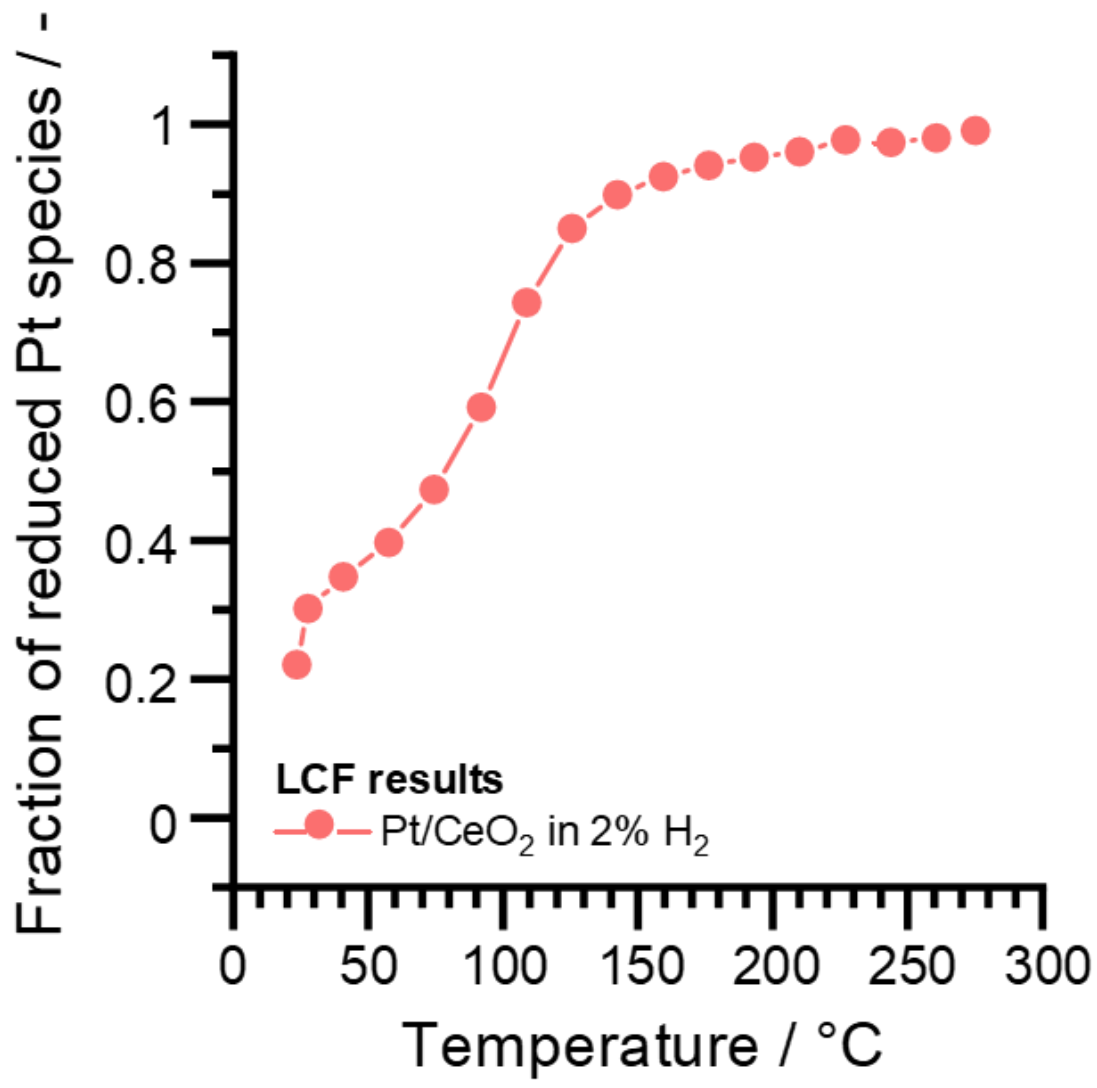

Figure S3. Results of the linear combination fitting (LCF) of the X-ray absorption near edge structure (XANES) spectra of Pt/CeO_2 cubes during the temperature programmed reduction (TPR) in 2% H_2/He. Before the TPR, the sample was calcined in air at 400 °C in 10 % O_2/He. For the linear combination fitting the state after oxidation and the last spectrum during the TPR were used as references.

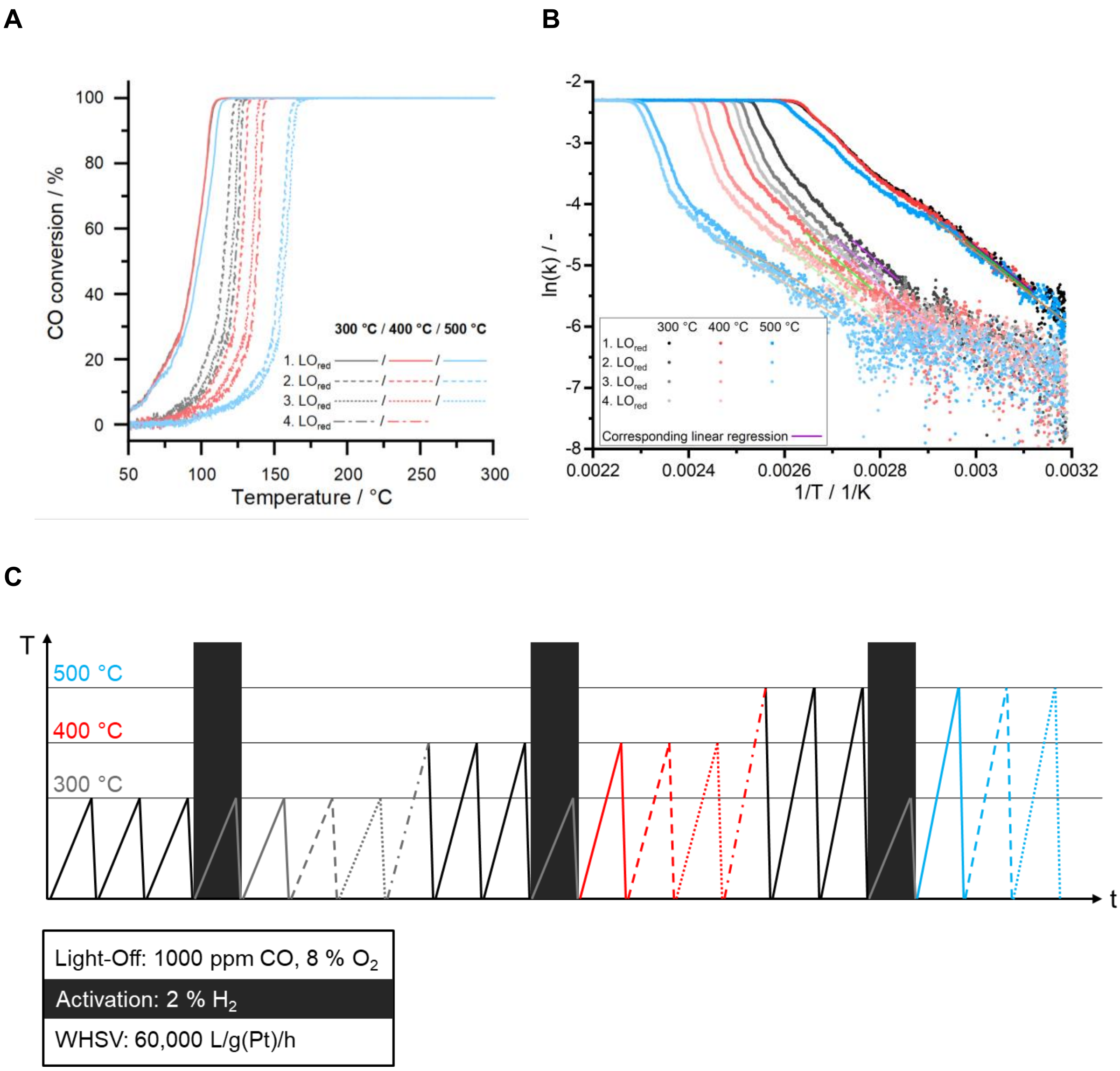

Figure S4. (A) CO conversion curves and (B) corresponding Arrhenius plots and fits during heating (light-offs) of Pt/CeO_2 cubes in a lean CO oxidation reaction mixture (1000 ppm CO, 8 % O_2 in N_2, 60,000 L/g_{Pt}/h). Prior to the tests, the catalyst was undergoing a reduction (2 % H_2 in N_2, 60,000 L/g_{Pt}/h) for 30 min at 300 °C. *1. LO_{red}*, *2. LO_{red}*, *3. LO_{red}*, *4. LO_{red}* corresponds to the first (straight lines), second (dashed lines), third (dotted lines) or fourth (dotted/dashed lines) light-off after the reduction, respectively. The colors of the lines indicate the maximum temperature reached during the light-off and also the holding temperature, at which the catalyst kept for 1 h in between each light off. The complete procedure is given as scheme (C).

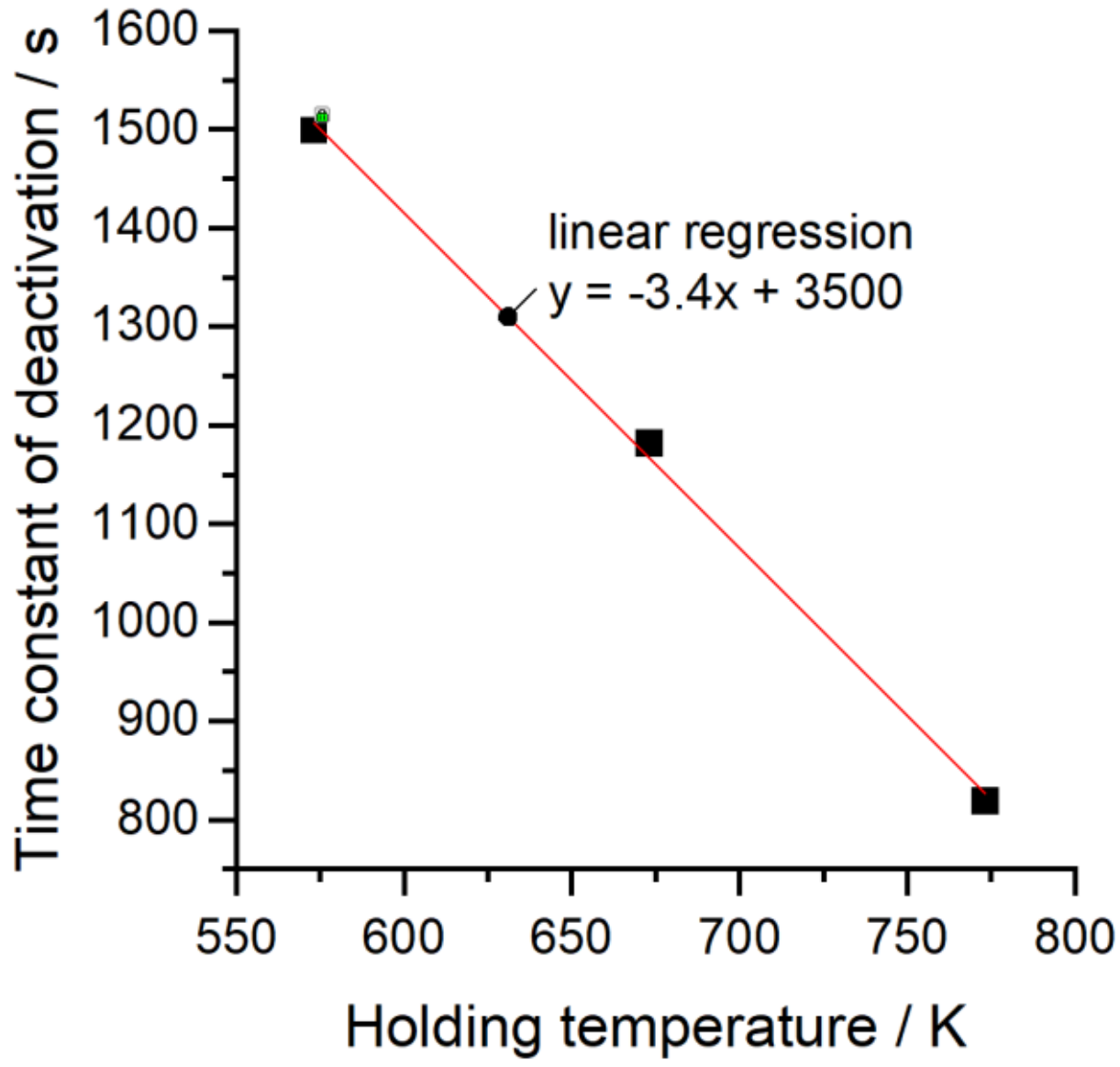

Figure S5. Time constant of deactivation obtained by the exponential fit in Fig. 5D against the holding temperature of deactivation. In red, a linear regression is performed showing a presumably linear dependency in the temperature window of 300 °C to 500 °C for the redispersion between both.

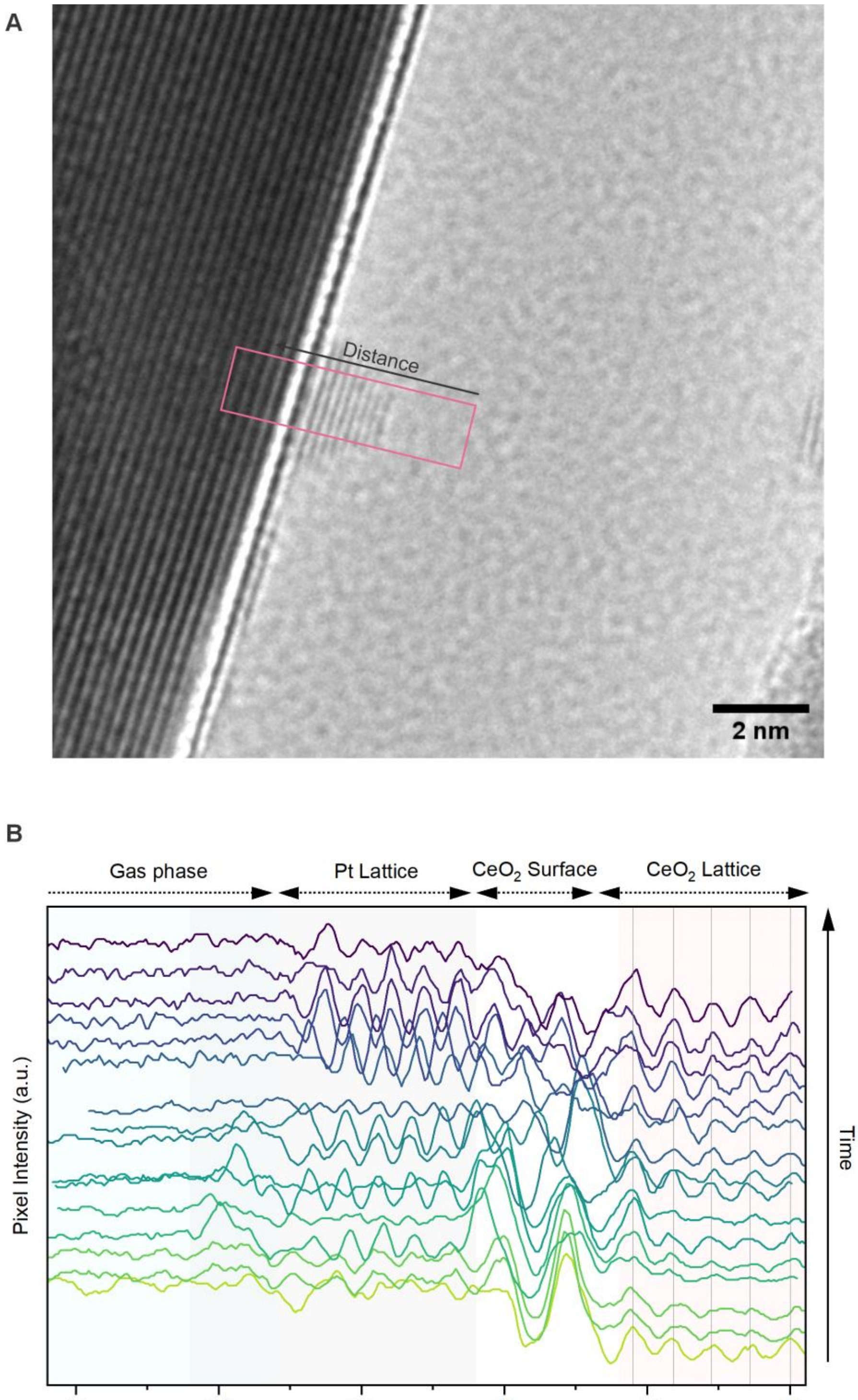

Figure S6. (A) ETEM image of a Pt nanoparticle on a CeO_2 cube. (B) Pixel intensity as function of distance from the gas phase at different time points during the Pt nanoparticle redispersion under oxidizing conditions. The CeO_2 lattice remains constant during the treatment enabling to evaluate even small expansions and contractions of the interplanar Pt-Pt lattice spacing during the redispersion (depicted in Fig. 4).

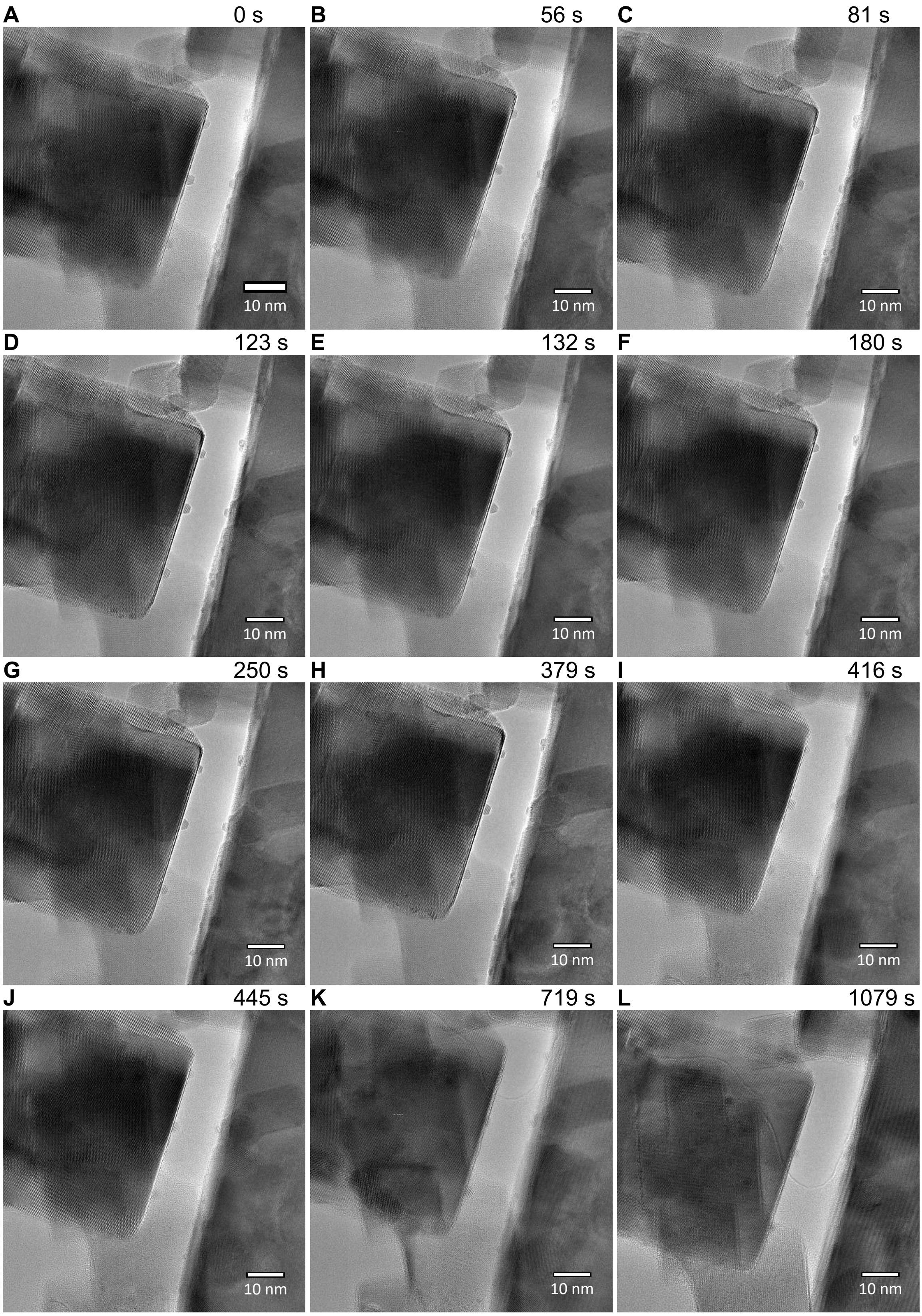

Figure S7. Series of ETEM images of Pt nanoparticles on CeO_2 cubes during redispersion at 500 °C and in 1.5 mbar O_2 in the time frame 0 s to 1079 s (A-L).

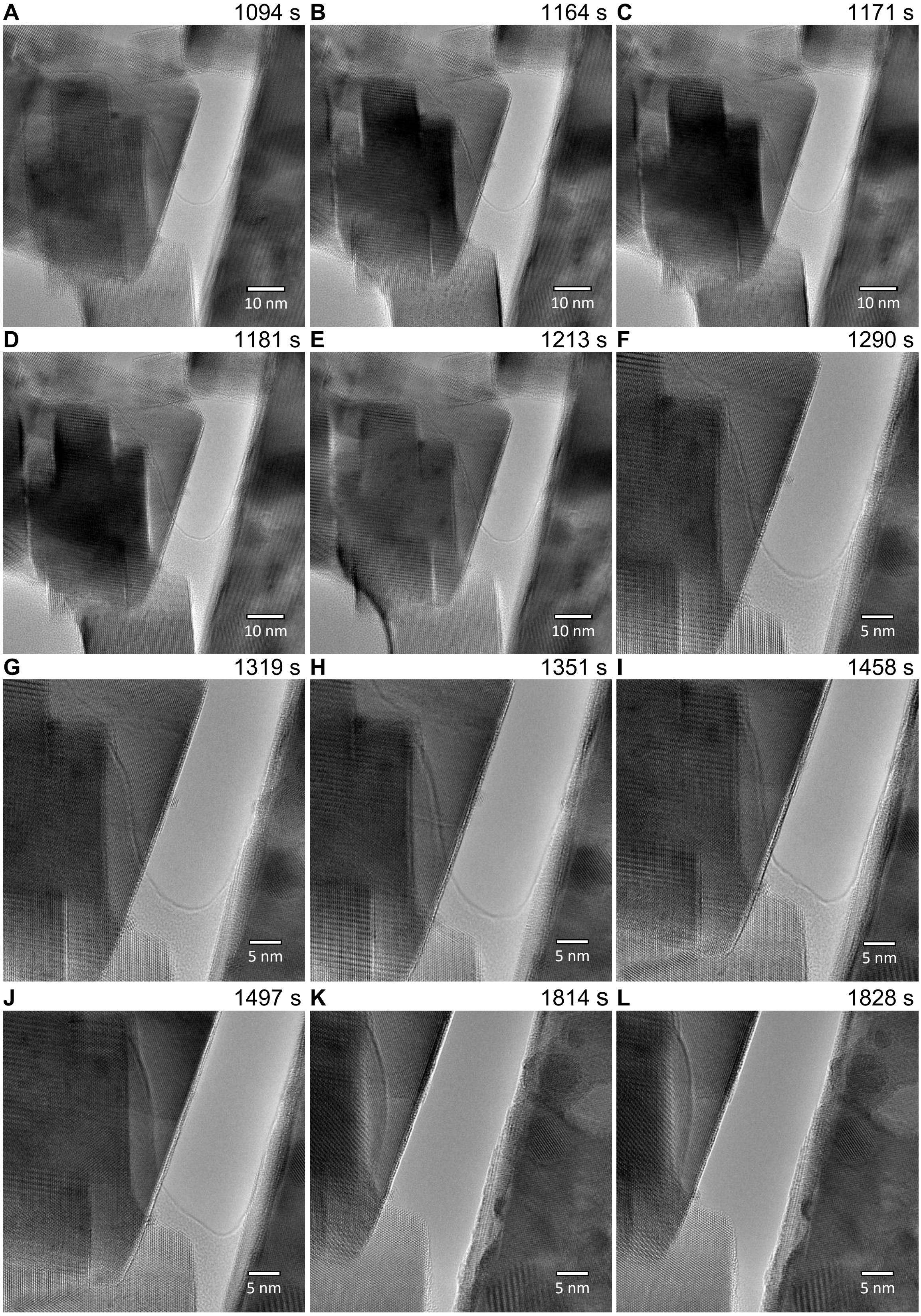

Figure S8. Series of ETEM images of Pt nanoparticles on CeO_2 cubes during redispersion at 500 °C and in 1.5 mbar O_2 in the time frame 1094 s to 1828 s (A-L).

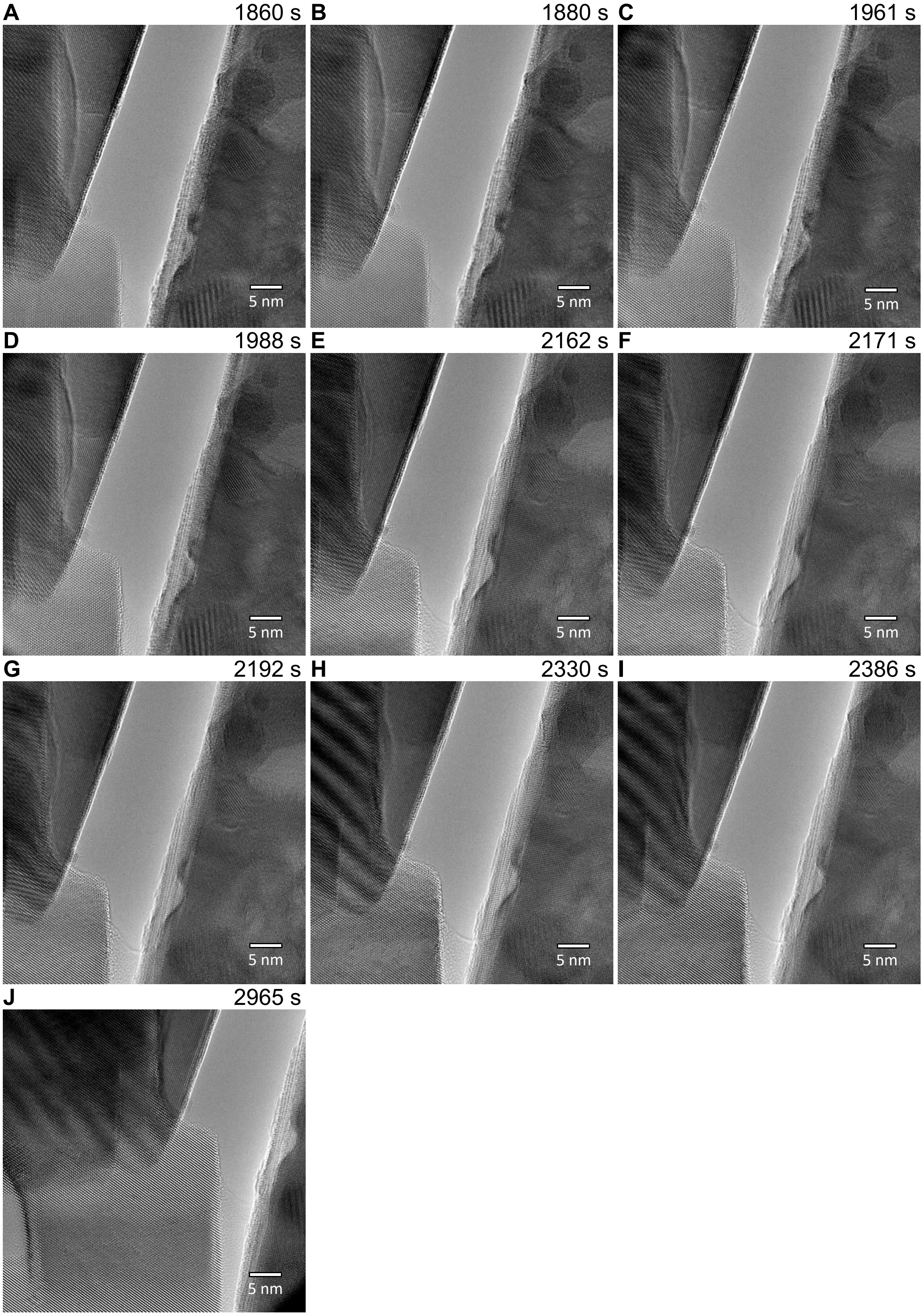

Figure S9. Series of TEM images of Pt nanoparticles on CeO_2 cubes during redispersion at 500 °C and in 1.5 mbar O_2 in the time frame 1860 s to 2965 s (A-J).

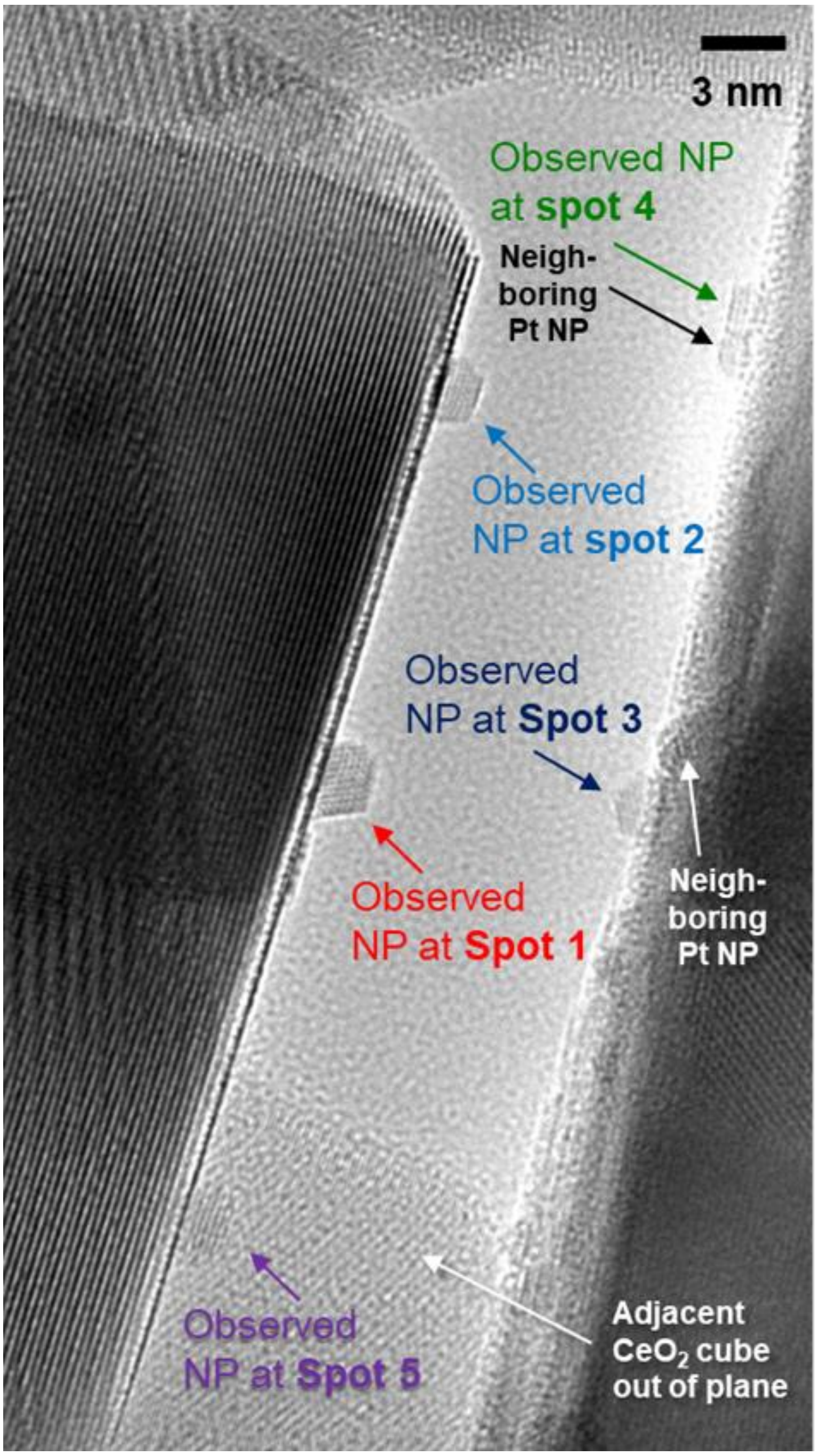

Figure S10. ETEM before the redispersion experiment at 500 °C in 1.5 mbar O_2. Arrows mark the observed Pt Nanoparticles at spots 1-5 as well as irregularities at spots 3-5 (black and with arrows).

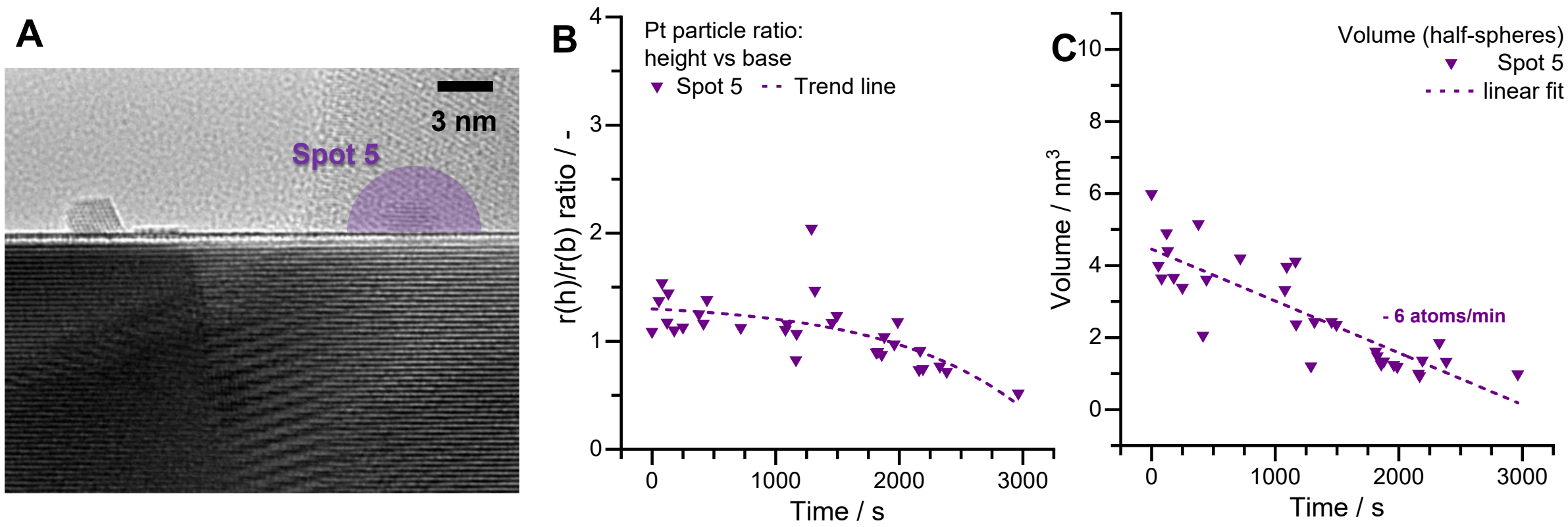

Figure S11. The TEM image (A) shows the region of interest with a fifth Pt NP highlighted in purple, which is overlapped by an adjacent CeO_2 cube. Analogously to the Figure 3, the time-dependent height-to-base ratio (B) and rate of redispersion (C) are given at 500 °C in 1.5 mbar O_2.

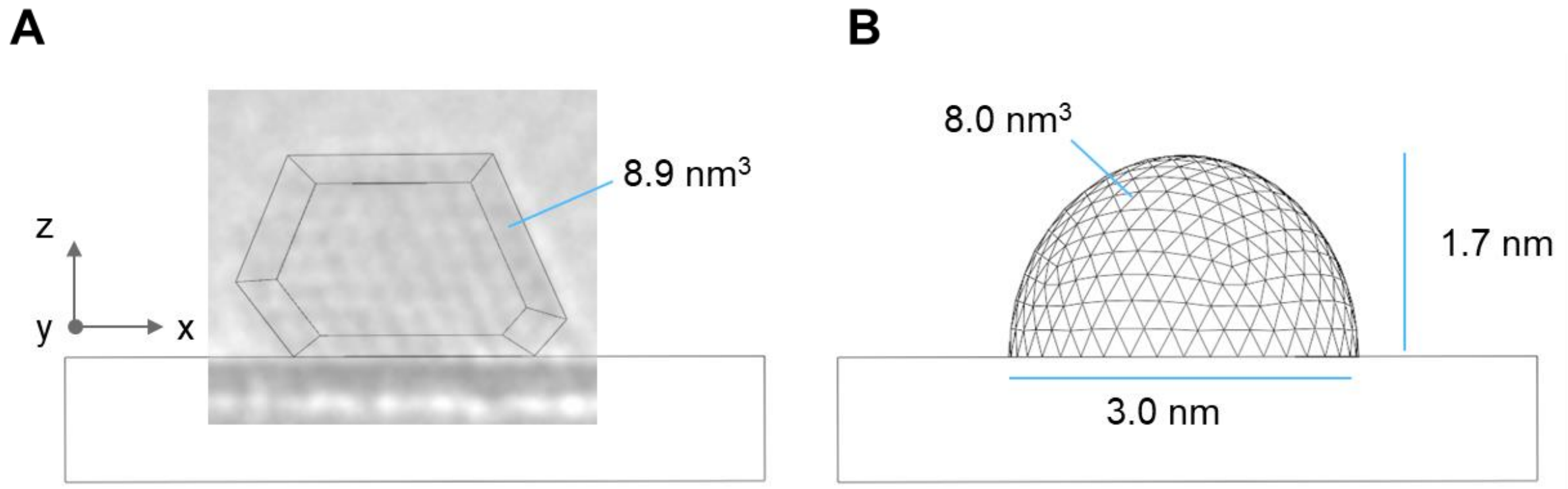

Figure S12. Estimation of the volume of the Pt nanoparticle at spot 1 and t=0 min using a faceted reconstruction assuming y-axis symmetry and the same particle length in x and y axis (A) and a simplified hemispherical model based on the height- and base-diameter (B).

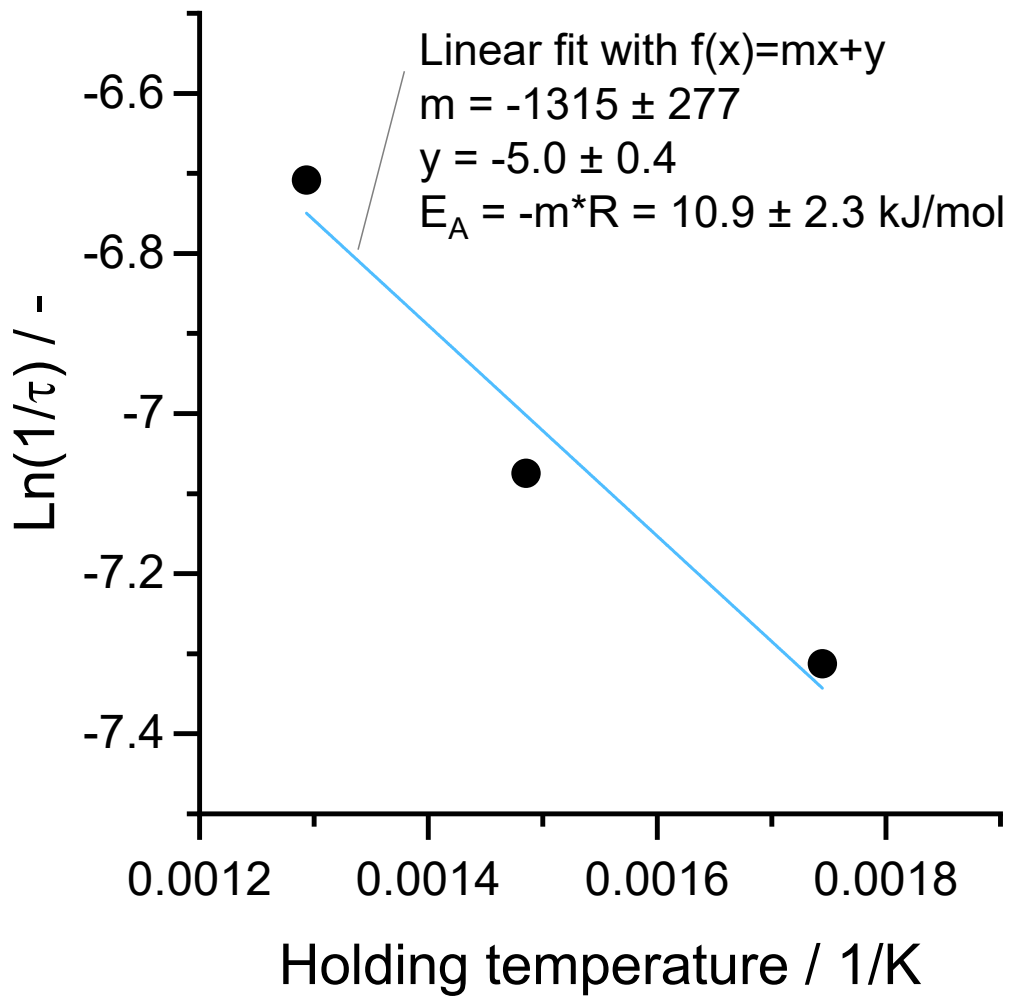

Figure S13. Estimation of the activation energy for the Pt redispersion under the assumption of the inverse proportionality of the activation decay time constants at 300 °C, 400 °C and 500 °C using the Arrhenius equation.

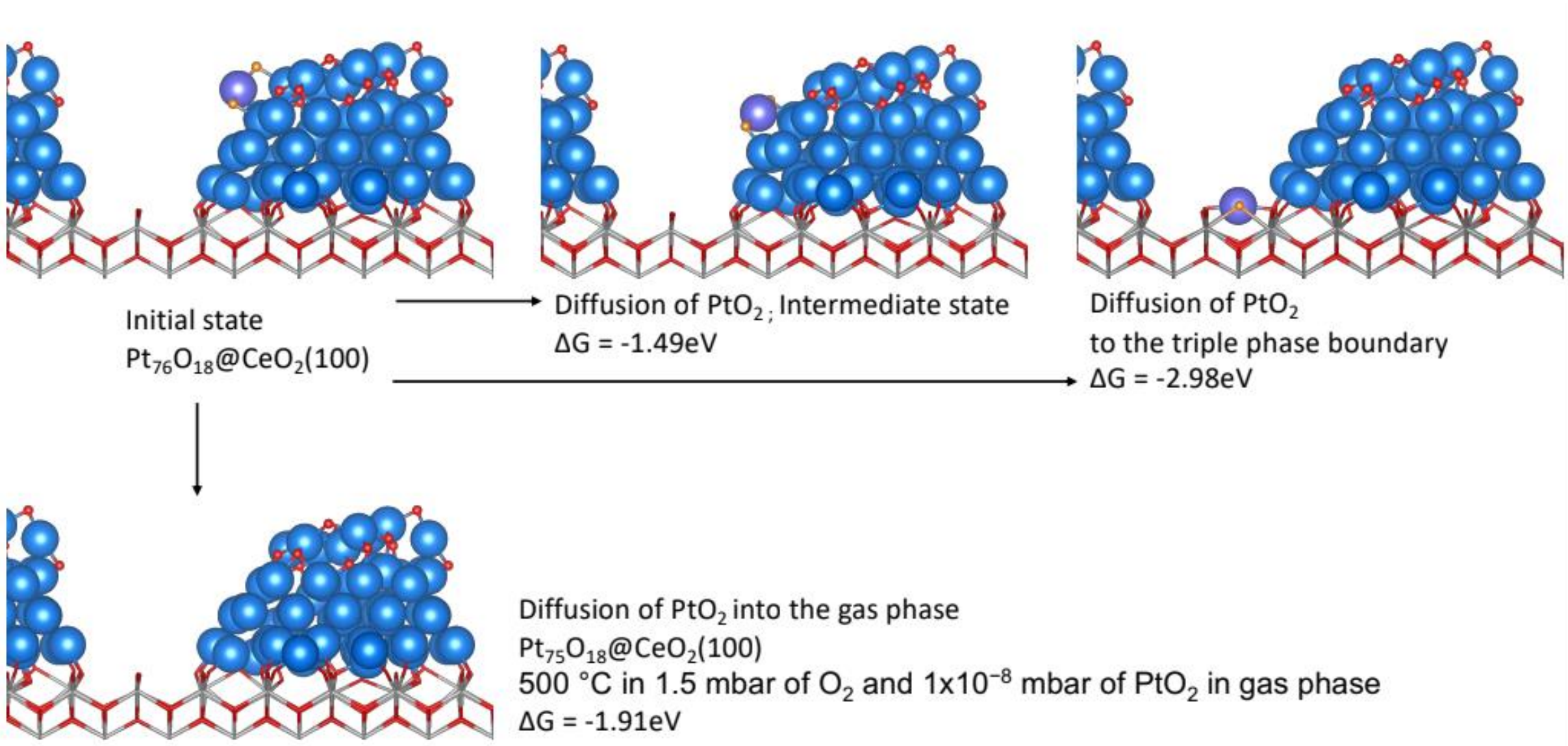

Figure S14. DFT-optimized structures of $Pt_{76}O_{18}$ on ceria considering the diffusion of a PtO_2 unit highlighted using purple instead of blue for Pt and orange instead of red for oxygen.

References

(1) Mai, H. X.; Sun, L. D.; Zhang, Y. W.; Si, R.; Feng, W.; Zhang, H. P.; Liu, H. C.; Yan, C. H. Shape-Selective Synthesis and Oxygen Storage Behavior of Ceria Nanopolyhedra, Nanorods, and Nanocubes. *J. Phys. Chem. B* **2005**, *109* (51), 24380–24385. https://doi.org/10.1021/jp055584b.

(2) Brunauer, S.; Emmett, P. H.; Teller, E. Adsorption of Gases in Multimolecular Layers. **1938**.

(3) Schindelin, J.; Arganda-Carreras, I.; Frise, E.; Kaynig, V.; Longair, M.; Pietzsch, T.; Preibisch, S.; Rueden, C.; Saalfeld, S.; Schmid, B.; Tinevez, J. Y.; White, D. J.; Hartenstein, V.; Eliceiri, K.; Tomancak, P.; Cardona, A. Fiji: An Open-Source Platform for Biological-Image Analysis. *Nat. Methods 2012 97* **2012**, *9* (7), 676–682. https://doi.org/10.1038/nmeth.2019.

(4) Abdala, P. M.; Safonova, O. V.; Wiker, G.; Beek, W. Van; Emerich, H.; Bokhoven, J. A. V.; Sá,

J.; Szlachetko, J.; Nachtegaal, M. Scientific Opportunities for Heterogeneous Catalysis Research at the SuperXAS and SNBL Beam Lines. *Chimia (Aarau).* **2012**, *66* (9), 699. https://doi.org/10.2533/chimia.2012.699.

(5) Bugaev, A.; Clark, A. H.; Genz, N. S.; Safonova, O. V.; Smolentsev, G.; Nachtegaal, M. X-Ray Spectroscopy at the SuperXAS and Debye Beamlines: From in Situ to Operando. *Chimia (Aarau).* **2024**, *78* (5), 304–312. https://doi.org/10.2533/chimia.2024.304.

(6) Clausen, B. S.; Steffensen, G.; Fabius, B.; Villadsen, J.; Feidenhans'l, R.; Topsøe, H. In Situ Cell for Combined XRD and On-Line Catalysis Tests: Studies of Cu-Based Water Gas Shift and Methanol Catalysts. *J. Catal.* **1991**, *132* (2), 524–535. https://doi.org/10.1016/0021-9517(91)90168-4.

(7) Ravel, B.; Newville, M. Synchrotron Radiation ATHENA, ARTEMIS, HEPHAESTUS: Data Analysis for X-Ray Absorption Spectroscopy Using IFEFFIT. *J. Synchrotron Rad* **2005**, *12*, 537–541. https://doi.org/10.1107/S0909049505012719.

(8) Kresse, G.; Hafner, J. Ab Initio Molecular Dynamics for Liquid Metals. *Phys. Rev. B* **1993**, *47* (1), 558–561. https://doi.org/10.1103/PhysRevB.47.558.

(9) Kresse, G.; Furthmüller, J. Efficient Iterative Schemes for Ab Initio Total-Energy Calculations Using a Plane-Wave Basis Set. *Phys. Rev. B* **1996**, *54* (16), 11169–11186. https://doi.org/10.1103/PhysRevB.54.11169.

(10) Perdew, J. P.; Ernzerhof, M.; Burke, K. Rationale for Mixing Exact Exchange with Density Functional Approximations. *J. Chem. Phys.* **1996**, *105* (22), 9982–9985. https://doi.org/10.1063/1.472933.

(11) Grimme, S.; Antony, J.; Ehrlich, S.; Krieg, H. A Consistent and Accurate Ab Initio Parametrization of Density Functional Dispersion Correction (DFT-D) for the 94 Elements H-Pu. *J. Chem. Phys.* **2010**, *132* (15). https://doi.org/10.1063/1.3382344.

(12) Grimme, S.; Ehrlich, S.; Goerigk, L. Effect of the Damping Function in Dispersion Corrected Density Functional Theory. *J. Comput. Chem.* **2011**, *32* (7), 1456–1465. https://doi.org/10.1002/jcc.21759.

(13) Kresse, G.; Joubert, D. From Ultrasoft Pseudopotentials to the Projector Augmented-Wave Method. *Phys. Rev. B* **1999**, *59* (3), 1758–1775. https://doi.org/10.1103/PhysRevB.59.1758.

(14) Blöchl, P. E. Projector Augmented-Wave Method. *Phys. Rev. B* **1994**, *50* (24), 17953–17979. https://doi.org/10.1103/PhysRevB.50.17953.

(15) Dudarev, S. L.; Botton, G. A.; Savrasov, S. Y.; Humphreys, C. J.; Sutton, A. P. Electron-Energy-Loss Spectra and the Structural Stability of Nickel Oxide: An LSDA+U Study. *Phys. Rev. B* **1998**, *57* (3), 1505–1509. https://doi.org/10.1103/PhysRevB.57.1505.

(16) Salichon, A.; Salcedo, A.; Michel, C.; Loffreda, D. Theoretical Study of Structure Sensitivity on Ceria-supported Single Platinum Atoms and Its Influence on Carbon Monoxide Adsorption. *J. Comput. Chem.* **2024**, *45* (25), 2167–2179. https://doi.org/10.1002/jcc.27393.

(17) Nolan, M.; Parker, S. C.; Watson, G. W. The Electronic Structure of Oxygen Vacancy Defects at the Low Index Surfaces of Ceria. *Surf. Sci.* **2005**, *595* (1–3), 223–232. https://doi.org/10.1016/j.susc.2005.08.015.

(18) Salcedo, A.; Alexandrova, A. N.; Loffreda, D.; Michel, C. Role of Oxidizing Conditions in the Dispersion of Supported Platinum Nanoparticles Explored by Ab Initio Modeling. *J. Phys. Chem. C* **2024**, *128* (9), 3805–3814. https://doi.org/10.1021/acs.jpcc.4c00355.
